# GABA_B_ Receptor signaling in CA1 Pyramidal Cells is not Regulated by Aging in the APP/PS1 Mouse Model of Amyloid Pathology

**DOI:** 10.1523/ENEURO.0099-23.2025

**Published:** 2026-02-05

**Authors:** Soraya Meftah, Max A. Wilson, Jamie Elliott, Lauren McLay, Vladimirs Dobrovolskis, Samuel Rosencrans, Lewis W. Taylor, Claudia Mugnaini, Rafaela Mostallino, Claire S. Durrant, Sam A. Booker

**Affiliations:** ^1^Centre for Discovery Brain Sciences, University of Edinburgh, Edinburgh EH8 9XD, United Kingdom; ^2^UK Dementia Research Institute (UK DRI), University of Edinburgh, Edinburgh EH16 4SB, United Kingdom; ^3^Simons Initiative for the Developing Brain, University of Edinburgh, Edinburgh EH8 9XD, United Kingdom; ^4^Dip. di Biotecnologie, Chimica e Farmacia, Università degli Studi di Siena, Siena 53100, Italy; ^5^Department of Biomedical Sciences, University of Cagliari, Monserrato 09042, Italy; ^6^Patrick Wild Centre for Research into Autism, Fragile X Syndrome, and Intellectual Disabilities, University of Edinburgh, Edinburgh EH8 9XD, United Kingdom

**Keywords:** aging, Alzheimer’s disease, amyloid pathology, GABA_B_ receptors, hippocampus, pyramidal cells

## Abstract

Dementia-causing diseases, including Alzheimer's disease (AD), are one of the greatest health concerns facing the aging world population. A key feature of AD is excessive accumulation of amyloid-beta, leading to synapse and cell loss in brain structures, such as the hippocampus. This neurodegeneration is preceded by impaired neuron function, notably reduced synaptic inhibition. Metabotropic GABA_B_ receptors (GABA_B_Rs) may be modulated by amyloid precursor protein (APP) and are reported to be progressively lost from neuronal membranes of hippocampal pyramidal neurons. However, it remains unknown whether functional GABA_B_R-mediated signaling changes over aging and whether or not pharmacological intervention can prevent receptor loss. In this study, we combine electrophysiological and biochemical analysis of hippocampal neurons in the Amyloid Precursor Protein/Presenilin-1 (APP/PS1) mouse model of AD from acute brain slices and organotypic slice cultures prepared from male and female mice to determine if functional GABA_B_Rs are lost and the effect of pharmacological modulation. Overall, we found that GABA_B_R expression decreased with age, independent of genotype, with no evidence for postsynaptic GABA_B_R loss in CA1 pyramidal cells at any age. We did observe a genotype-dependent reorganization of postsynaptic GABA_B_R-mediated IPSCs, which was independent of age. Presynaptic GABA_B_R-mediated inhibition was impaired in APP/PS1 mice, also independent of age. We observed that chronic GABA_B_R modulation differentially regulated function but was independent of genotype. Overall, our data show that functional GABA_B_R signaling is altered in APP/PS1 mice, independent of age, increasing our understanding of amyloidopathy-induced dysfunction.

## Significance Statement

Alzheimer's disease (AD) is a currently incurable disease causing severe memory loss and leading to dementia. It is thought that a main cause of dementia is a disruption to synaptic between neurons, especially inhibitory connections that normally reduce neuron activity. Amyloid-beta (Aβ), a protein that accumulates in the brain during AD, is thought to damage inhibitory synapses, but the exact mechanism by which this happens is unknown. Using a mouse model of Aβ pathology, we will test whether GABA_B_ receptors activated by inhibitory synapses are disrupted in a model of AD and whether modulating their activity accelerates or slows the progression of disease-related phenotypes. These findings may elucidate novel dementia progression mechanisms and provide new therapeutic insights.

## Introduction

Alzheimer's disease (AD) and other forms of dementia are leading causes of death worldwide, contributing to ∼11% of deaths in the United Kingdom and up to 13.6% of deaths in the United States (Mesalles-Naranjo et al., 2018; Veitch et al., 2019). AD typically presents in later life (most commonly in individuals over the age of 65) with the main symptoms being extensive and progressive cognitive decline and loss of executive functions (Veitch et al., 2019). Dementia leads to a dramatic reduction in the quality of lives of those affected and their families and represents a significant economic burden (Takizawa et al., 2014; Tahami Monfared et al., 2022).

AD pathologies are thought to arise from alterations in the physical state and function of amyloid-beta (Aβ) and tau proteins, which ultimately results in synaptic dysfunction, neuroinflammation, and neuronal death (Kent et al., 2020; Tzioras et al., 2023). As repairing damaged synapses and neurons in the adult brain is likely to be extremely challenging, our priority should be to identify the earliest pathological changes that occur in AD, such that we can develop therapeutic approaches to prevent future synapse loss. Changes in Aβ are thought to arise upstream of changes to tau, and so understanding exactly how such early changes impact synaptic function is essential. Recent studies have examined the proteome of individuals with AD which identify alterations in the levels of many proteins involved in synaptic function, compared with unaffected controls (Hesse et al., 2019), particularly those that relate to inhibitory neurotransmission in the hippocampus and neocortex. The APP/PS1 mouse, a well-characterized model of Aβ pathology, has been shown to recapitulate many aspects of AD progression seen in affected humans, including cognitive impairment, Aβ plaque deposition, synapse loss (Trinchese et al., 2004; Yan et al., 2009; Viana da Silva et al., 2016), and altered inhibitory signaling (Takahashi et al., 2010; Oyelami et al., 2016; Hollnagel et al., 2019). Understanding the earliest mechanisms that lead to altered neurotransmission in this model may be of great value in identifying effective therapeutics against Aβ toxicity, especially those that may work as adjuncts alongside Aβ-removal strategies, such as lecanemab and donanemab (Sims et al., 2023; Van Dyck et al., 2023).

Mature hippocampal circuits comprised of excitatory pyramidal cells and inhibitory GABAergic interneurons (INs) mediate the flow of synaptic information (Booker and Vida, 2018), the appropriate balance of which allows for memory storage and retrieval (Topolnik and Tamboli, 2022). Excitatory neurons and the synapses they form display a well-described pattern of decline and loss in the APP/PS1 mouse model of Aβ pathology. Specifically, in late adulthood (>6 months of age), excitatory synapses display structural modification and synapse loss associated with accumulation of Aβ (Knafo et al., 2009; Alonso-Nanclares et al., 2013). Such processes are present in organotypic slice culture (OSC) preparations, albeit over an accelerated timescale (Harwell and Coleman, 2016; Durrant, 2020). In parallel with this, local INs also display cell and synapse loss (Ramos et al., 2006; Takahashi et al., 2010; Schmid et al., 2016; Hijazi et al., 2020; Gervais et al., 2022), which is paralleled in the brains of AD patients (Waller et al., 2020). GABA released within the local circuit acts on fast, ionotropic GABA_A_ receptors (GABA_A_Rs) and slow, metabotropic GABA_B_ receptors (GABA_B_Rs)—the latter of which is primarily activated by auto- and heterosynaptic spillover of GABA (Scanziani, 2000; Booker et al., 2013; Watson and Booker, 2024). Both GABA_A_Rs and GABA_B_Rs have been implicated in AD (Chu et al., 1987), but GABA_B_Rs appear to be involved in a more pronounced, progressive, and cell-wide manner (Salazar et al., 2021); especially in pyramidal cells of the hippocampus (Martín-Belmonte et al., 2020a,b, 2022).

GABA_B_Rs act postsynaptically on neurons by activating G-protein–coupled inward-rectifying K^+^ (GIRK/Kir3) channels to hyperpolarize their membranes and also inhibit voltage-gated Ca^2+^ channels (VGCCs; Pinard et al., 2010). At presynaptic axon terminals, GABA_B_Rs inhibit VGCCs to prevent exocytosis of neurotransmitters. These mechanisms exist on both pyramidal cells and INs to control their activity (Kulik et al., 2018) in a synapse- and compartment-specific manner. Amyloid precursor protein (APP), which is cleaved to form Aβ, has been suggested to act as modulator of GABA_B_R function (Dinamarca et al., 2019) and contentiously as an endogenous agonist (Rice et al., 2019; Rem et al., 2023). In response to sustained activation, GABA_B_Rs rapidly desensitize and then internalize, leading to reduced functional currents (Turecek et al., 2014; Li et al., 2020). This raises the possibility that sustained activation of GABA_B_Rs, supported through allosteric modulation by APP, may lead to the reduced expression profile of GABA_B_Rs in AD (Martín-Belmonte et al., 2020a,b, 2022). As such, it is plausible that administration of competitive antagonists (i.e., CGP-55,845; Brugger et al., 1993) or negative allosteric modulators (NAM; e.g., COR758; Porcu et al., 2021) may reduce excessive GABA_B_R activation, which in turn may prevent receptor loss. While such a therapeutic route is (to the best of our knowledge) untested in AD, proof of principle for cognitive benefit has been shown in typical rat aging (LaSarge et al., 2009).

We hypothesize that functional GABA_B_R signaling is reduced in the APP/PS1 mouse model of AD in a developmental and synapse-specific manner, and this loss may be prevented by negative modulation of GABA_B_R activity. This hypothesis will be addressed by the following aims: confirm that Aβ pathology in the APP/PS1 mouse model leads to a reduction of the functional GABA_B_R-mediated responses in hippocampal pyramidal cells; define the developmental trajectory of presynaptic GABA_B_R control of neurotransmitter release in a synapse-specific manner and how this is impacted by Aβ pathology; determine if the hippocampal network is less sensitive to GABA_B_R modulation in the aging APP/PS1 mouse brain; and determine whether inhibition of GABA_B_Rs can prevent functional receptor loss and alter circuit function in the APP/PS1 mouse model of dementia.

To achieve these aims, we employed single-cell and population-level electrophysiological recordings from acute brain slices from juvenile (1-month-old), adult (6-month-old), and aged (1-year-old) APP/PS1 mice (both male and female). To allow direct pharmacological and genetic manipulation, we generated OSCs from APP/PS1 mice to determine whether modulation of GABA_B_R activation contributes to changes in this receptor signaling cascade. These findings are supported and extended by biochemical assays for perisynaptic expression of the GABA_B_R signaling pathway over age and in response to genotype.

## Materials and Methods

### Animals

All animals were obtained from transgenic breeding from heterozygous APP/PS1 (Mo/HuAPP695swe/PS1-dE9) crossed to WT (C57/BL6J) mice, to give 1:1 WT/transgenic offspring, and both male and female mice were used for experiments. For acute brain slice experiments, mice were taken at 1, 6, or 12 months of age. For OSCs, mice were taken at 6–9 d old. All experiments were performed in accordance with institutional (University of Edinburgh) and UK Home Office guidelines (ASPA; PPL, PCB113BFD and PP8710936). All mice were maintained in 12 h light/dark cycles, housed in littermate groups in cages of 4–6 mice, and given *ad libitum* access to food and water.

### Organotypic brain slice preparation

OSCs were prepared as described previously (Durrant, 2020; Taylor et al., 2024). Briefly, mouse pups aged Postnatal Day 6–9 were humanely culled by cervical dislocation followed by decapitation. The brain was then rapidly removed and transferred to ice-cold “dissection medium” (in mM: 87 NaCl, 2.5 KCl, 25 NaHCO_3_, 1.25 NaH_2_PO_4_, 25 glucose, 75 sucrose, 7 MgCl_2_, 0.5 CaCl_2_, 1 Na-pyruvate, 1 Na-ascorbate, 1 kynurenic acid; 100 U/ml penicillin/streptomycin) bubbled with carbogen on ice. Brains were then glued using cyanoacrylate onto a Leica VT1200S vibratome stage, and submerged in ice-cold, carbogenated “dissection medium.” Horizontal sections (350 µm thick) were taken from the hippocampus, at a speed of 0.33 mm/s and 1.8 mm amplitude. Hippocampal sections were dissected out using a fine needle to obtain horizontal hippocampal sections with a small section of the entorhinal cortex attached. Between 6 and 8 hippocampal slices were obtained per pup. Slices (two per dish) were then plated, using a 1 ml Pasteur pipette, onto membranes (Millipore PICM0RG50) in 35 mm dishes with 1 ml of “maintenance medium” [MEM with GlutaMAX-1 (50%; Invitrogen, 42360032), heat-inactivated horse serum (25%; Thermo Fisher Scientific, 26050070), EBSS (18%; Thermo Fisher Scientific, 24010043), d-glucose (5%; Sigma-Aldrich, G8270), 1× penicillin/streptomycin (Thermo Fisher Scientific, 15140122), nystatin (3 U/ml; Merck, N1638), and ascorbic acid (500 μM; Sigma-Aldrich, A4034)] underneath the insert. Maintenance medium was filtered through a 0.22 µm filter prior to use. Slices were maintained in an incubator at 37°C, 5% CO_2_ and 100% humidity for 1–2 months. Slices underwent 100% medium changes within the first 24 h, twice within the first week (∼Day 4 and Day 7), and then fed weekly thereafter. Cultures from APP/PS1 pups and WT littermates were assessed for changes in functional GABA_B_R signaling in vitro. Treatments were applied for 1–2 weeks at 4 weeks in culture to assess the impact of GABA_B_R inhibition on electrophysiological function in APP/PS1 and WT littermates.

### Acute brain slice preparation

Acute brain slices containing the hippocampus were prepared as previously described (Oliveira et al., 2021). Briefly, mice were terminally anesthetized with isoflurane, decapitated, and their brain dissected into semifrozen sucrose–artificial cerebrospinal fluid (ACSF; in mM: 87 NaCl, 2.5 KCl, 25 NaHCO_3_, 1.25 NaH_2_PO_4_, 25 glucose, 75 sucrose, 7 MgCl_2_, 0.5 CaCl_2_, 1 Na-pyruvate, 1 Na-ascorbate) bubbled with carbogen. Horizontal brain slices (300 μm thick) were cut on a Vibratome (VT1200s, Leica Biosystems) in semifrozen sucrose–ACSF and then stored submerged in sucrose–ACSF warmed to 35°C for 30 min and subsequently at room temperature.

### Whole-cell patch–clamp recordings

For whole-cell patch–clamp recordings, acute slices or slice cultures were transferred to a submerged recording chamber supplied with carbogenated recording ACSF (in mM: 125 NaCl, 2.5 KCl, 25 NaHCO_3_, 1.25 NaH_2_PO_4_, 25 glucose, 1 MgCl_2_, 2 CaCl_2_), at 5–6 ml/min at 31 ± 1°C by an inline heater. Slices were then visualized under Köhler illumination by means of an upright microscope (SliceScope, Scientifica), equipped with a 40× water-immersion objective lens (N.A. 0.8; Olympus). Hippocampal CA1 pyramidal cells were identified as ovoid cells located in stratum (str.) pyramidale or upper str. oriens. Whole-cell patch–clamp recordings were amplified using a Multiclamp 700B amplifier (Molecular Devices). Recording pipettes were made from borosilicate glass capillaries (1.5 mm outer/0.86 mm inner diameter, Harvard Apparatus) on a horizontal electrode puller (P-97 or P-1000, Sutter Instruments). When filled with intracellular solution (in mM: 142 K-Gluc, 4 KCl, 2 MgCl_2_, 0.1 EGTA, 10 HEPES, 2 Na_2_-ATP, 0.3 Na_2_-GTP, 10 Na_2_-phosphocreatine, 0.1% biocytin, 290–310 mOsm), pH 7.35, this gave pipette resistances of 2–6 MΩ. Unless otherwise stated, all voltage-clamp recordings were performed at a holding potential of −70 mV and all current-clamp recordings from the resting membrane potential (V_M_). For all recordings, series resistance (R_S_) was monitored but not compensated for in voltage clamp, and the bridge was balanced following pipette-capacitance compensation in current clamp. Signals were filtered online at 2–10 kHz using the built-in two–pole Bessel filter of both amplifiers, digitized and acquired at 20 kHz (Digidata 1550B, Axon Instruments), using pClamp 10 (Molecular Devices). Data were analyzed offline using the open-source Stimfit software package (Guzman et al., 2014; http://www.stimfit.org) or in MATLAB using custom code. The liquid junction potential of this recording configuration has been measured as ∼12 mV, but was not adjusted.

After obtaining a whole-cell recording, the intrinsic properties of recorded neurons were characterized in current-clamp mode from resting membrane potential. A family of 500 or 1,000 ms hyper- to depolarizing current steps (−500 to +500 pA, 100 pA steps or −100 to +350 pA, 50 pA steps, respectively) was used depending on the input resistance of the neuron. Cells were initially identified based on their voltage response and the resulting train of action potentials (APs) elicited by a family of hyper- to depolarizing current steps (50 pA, 500 ms duration; −500 to +500 pA). Neurons were rejected from further analysis if the resting membrane potential was more depolarized than −50 mV, APs failed to overshoot 0 mV, initial access resistance (R_A_) exceeds 30 MΩ, or R_A_ fluctuated by >20% over the time course of the experiment.

### Determination of postsynaptic GABA_B_R-mediated currents

Postsynaptic GABA_B_R-mediated currents were measured by using endogenous release of GABA and pharmacological characterization, as previously described (Booker et al., 2020; Watson and Booker, 2024). Pharmacologically isolated GABA_B_R-mediated currents were measured in the presence of ionotropic receptor blockers added to the perfusing ACSF: CNQX (10 µM), DL-AP5 (50 µM), and picrotoxin (50 µM). To evoke GABA_B_R-mediated inhibitory postsynaptic currents (IPSCs) and potentials (IPSPs), extracellular stimuli were delivered by a bipolar, twisted Ni:Chrome electrode. For CA1 pyramidal cells, stimuli elicited in *str. lacunosum-moleculare* (L-M), *radiatum*, and *oriens* was used to determine compartment-specific deficits in synaptic function (Booker et al., 2013). GABA_B_R-mediated IPSCs were evoked either by single stimuli or 200 Hz trains of five stimuli and recorded at −65 mV voltage clamp. Stimuli were delivered at 0.1 Hz and a minimum of 10 IPSCs collected for each pharmacological epoch or subcellular compartment. In a subset of cells, IPSPs were recorded in current clamp from resting membrane potential (V_M_). Then V_M_ was shifted over a range of potentials (from −50 to −120 mV), via application of bias current, to assess the reversal potential (E_R_) of the IPSPs. To assess the whole-cell currents (*I*_WC_) mediated by GABA_B_Rs, we bath applied the orthosteric GABA_B_R agonist *R*-baclofen (10 µM) for 10 min, followed by bath application of the selective antagonist CGP-55,845 (CGP; 5 μM). The amplitude of GABA_B_R-mediated IPSCs was measured from average traces of each pharmacological epoch, from a minimum of 10 traces. Peak amplitude was measured over a 10 ms window within 200 ms of the last stimulus, measured relative to the prestimulus baseline. Kinetics of GABA_B_R-mediated IPSCs were measured as 20–80% rise time, decay-time constant from a monoexponential curve fit, and total conductance (IPSC integral), all of which were measured from the prestimulus baseline. Baclofen I_WC_ was measured as both the peak current (30 s/3 trace average) and the steady-state current measured following 9–10 min of *R*-baclofen application. The desensitization ratio (Booker et al., 2017) of GABA_B_R *I*_WC_ in response to *R*-baclofen was measured as follows:

$$\mathrm{Densensitization}\;\mathrm{ratio}\;=\;(\mathrm{Peak}\;I_{\mathrm{WC}}-\;\mathrm{SteadyState}\;I_{\;\mathrm{WC}})/\mathrm{Peak}\;I_{\mathrm{WC}}.$$

The relative effect of CGP on *I*_WC_ was assessed as the average current recorded over 4–5 min following bath wash-in.

### Determination of presynaptic GABA_B_R-mediated inhibition

For presynaptic characterization of GABA_B_R-mediated signaling, neurons were recorded as previously described (Booker et al., 2020; O’Keeffe et al., 2025), with a cesium gluconate-based internal solution (in mM: 140 Cs-Gluc, 3 CsCl, 0.2 EGTA, 10 HEPES, 2 Na_2_-ATP, 2 Mg-ATP, 0.3 Na_2_-GTP, 10 Na_2_-phosphocreatine, 5 QX-314.Cl, 0.1% biocytin, 290–310 mOsm), pH 7.35, to block any postsynaptic K^+^ conductance and improve voltage-clamp conditions. Slices were transferred to the recording chamber, which was perfused with recording ACSF (no pharmacology). In all recordings, spontaneous EPSCs (sEPSCs) were recorded from −70 mV voltage clamp (*E*_R[Cl−]_ = −74 mV), and then IPSCs were recorded from ∼0 mV voltage clamp (*E*_R[AMPAR/NMDAR]_ = 0 mV) for 2 min prior to stimulation to determine the basal synaptic inputs to recorded neurons, the same spontaneous recording was performed under all pharmacological epochs. For hippocampal recordings, bipolar stimulating electrodes were placed in both *str. L-M* and *str. radiatum*, and then a CA1 pyramidal cell was recorded in whole-cell configuration. Synaptically evoked EPSCs were then recorded at −70 mV and stimuli recorded in response to increasing stimulus intensity (0–25 V, 5 V increments), after which an EPSC amplitude of 100–200 pA was selected for pharmacological assay. Pairs of electrical stimuli (50 ms interval) were delivered to both pathways independently under basal conditions. A minimum of 60 traces were recorded for each epoch at 0.2 Hz. Following this baseline, 10 µM *R*-baclofen was applied to the bath and 5 min of response recorded. To confirm the GABA_B_R specificity of this response, we applied 5 μM CGP for a further 5 min. For synaptic-evoked IPSCs, the recording setup was identical; except those cells were held at 0 to +10 mV (*E*_R[AMPAR/NMDAR]_ ∼ 0 mV).

The amplitude of PSCs was measured as the peak (from prestimulus baseline) of each PSC (0.5 ms average) over 0–10 ms following the stimuli. The first and second PSCs were measured to calculate the paired pulse ratio (PSC_2_/PSC_1_). For determination of pharmacological effects, the PSC amplitude was compared before and after each drug wash-in. To determine the synaptic innervation of neurons, we analyzed the frequency, amplitudes, and kinetics of properties of spontaneous PSCs recorded under each epoch. To achieve this, we used a template matching detection criteria (Clements and Bekkers, 1997) based on a triexponential fit to examples responses. Events were included for analysis if they were three times the standard deviation of the baseline noise. The amplitude and frequency were calculated from individual extracted events, while the kinetics were measured from an all-event average trace.

### Oscillation analysis and local-field potentials

To ensure preservation of intact local networks, we sliced 400-µm-thick acute hippocampal slices (as above) in the transverse plane and then stored them in a liquid/gas interface chamber (Brown et al., 2007; Booker et al., 2020). For gamma oscillations, slices were transferred from the storage chamber, into an interface recording chamber perfused with ACSF at 32°C. For extracellular recordings, a recording electrode made from a patch pipette filled with ACSF was carefully placed in either *str. radiatum* or *str. L-M* and the local-field potential (LFP) recorded continuously. Following a 30 min equilibration, kainic acid (50 nM) and carbachol (2.5 µM) were added to the perfusing ACSF, and oscillations were allowed to develop, which normally takes 40–60 min. Once stable oscillations were detected for at least 10 min, we then bath applied baclofen (2 and 10 µM, 40 min each) to preferentially activate pre- and postsynaptic receptors, respectively (Booker et al., 2018). Finally, we bath applied the GABA_B_R antagonist CGP (5 μM) for a further 40 min to confirm receptor specificity. Prior to analysis, all field recordings were notch-filtered at 49–51 Hz to remove noise artifacts. The 2 min windows during the last 10 min of each pharmacological epoch were analyzed using fast Fourier transform (FFT)-based spectral analysis (Spike2 software, CED) to generate power spectra with a frequency resolution of 0.61 Hz. The power spectral density (μV/Hz) was calculated, and the oscillatory strength was quantified using the area under the curve (AUC) measure between 20 and 49 Hz.

### Synaptosome preparation

Synaptosomes were prepared as described previously for organotypic slices (Croft et al., 2017). Briefly, acute dissected hippocampi were homogenized in lysis buffer [in mM: 10 Tris–HCl, 320 sucrose, supplemented with complete EDTA-free protease inhibitor cocktail tablets and Halt Phosphotase Inhibitor Cocktail, 2 EDTA (Thermo Fisher Scientific 78429), 2 EGTA], pH 7.4, and centrifuged at 1,000 × *g* for 10 min at 4°C to remove cell nuclei. The supernatant was further centrifuged at 10,000 × *g* for 20 min at 4°C. The resulting final supernatant represents the nonsynaptosome fraction, while the pellet contained synaptosomes. All fractions were resuspended in 10 mM Tris–HCl and stored at −70°C until use.

### Western blot analysis of protein levels

Total homogenate or synaptosomes as generated above were diluted 50:50 in 2× Laemelli buffer then boiled for 10 min at 98°C. Five–twenty micrograms of protein was run per lane on a NuPAGE 4–12% Bis-Tris gel in SDS running buffer for 90 min at 120 V, 400 mA. Protein was transferred from the gel to a PVDF membrane using an iBlot 3 Dry blotting system, for 8.5 min at 20 V. A REVERT total protein stain was performed according to manufacturer's instructions to assess protein transfer. After imaging the total protein using a Licor Odyssey blot scanner, the REVERT stain was removed (de-stain procedure), and the membrane underwent 1 h of blocking using Intercept Blocking Buffer. After block, primary antibodies were incubated on the membrane overnight in blocking buffer +0.1% Tween-20 overnight at room temperature with gentle shaking. After washing three times with PBS +0.1% Tween-20 (PBS-T), Licor fluorescent secondary antibodies were added at 1:10,000 to blocking buffer and was incubated for 2 h at room temperature. After the final three washes in PBS-T, images were taken on the Licor Odyssey scanner, and proteins of interest were normalized to a general housekeeping protein (GAPDH, Abcam #AB9485). Samples were assessed for GABA_B_R_1_ (Millipore, AB2256; 115 kDa), Kir3.2 (Alomone Labs, APC-006-GP; ∼45 kDa), and synaptophysin (Abcam, ab8049, ∼38 kDa).

### Visualization, labeling, and reconstruction of recorded neurons

Reduced neuronal expression of pre- or postsynaptic GABA_B_Rs has been observed across the hippocampus of the APP/PS1 mouse model of dementia (Martín-Belmonte et al., 2020a,b, 2022); however, other studies have noted a correlation of cellular and synaptic phenotypes as proximity to Aβ plaques increases (Koffie et al., 2009). To address this potential source of variability in our data, we performed post hoc visualization of neurons following recording, combined with immunolabeling for Aβ (to ascertain plaque location).

All recorded neurons were visualized* post hoc* as previously described (Booker et al., 2014) to confirm that dendritic arbors were intact and to assess dendritic spine density. Briefly, following successful outside-out patch formation, slices were fixed in 4% paraformaldehyde in 0.1 M phosphate buffer (PB) overnight at 4°C. Slices were then washed in PB and then incubated with fluorescent-conjugated streptavidin (Alexa Fluor 633; 1:500, Invitrogen) in solution containing 0.5% Triton X-100 and 0.05% NaN_3_ at 4°C overnight. Slices were washed in PB. For Aβ immunolabeling, slices were blocked with 10% normal goat serum, 0.5% Triton X-100, and 0.05% NaN_3_ for 1 h and then incubated with mouse anti-Aβ (MOAB, clone 6C3, 1:1,000, Millipore) in 5% normal goat serum, 0.5% Triton X-100, and 0.05% NaN_3_ overnight. Slices were rinsed and secondary antibodies (goat anti-mouse Alexa Fluor 488) applied in a solution of 3% normal goat serum, 0.1% Triton X-100, and 0.05% NaN_3_ for 24 h and then washed in PBS. The slices were then washed liberally in PB and mounted on glass slides with a polymerizing mounting medium (Fluoromount-G, Southern Biotech) and stored in the dark until imaging.

CA1 pyramidal neurons were imaged using a confocal laser scanning microscope (Leica SP8, Olympus) equipped with a 20× (N.A. 0.75) air-immersion lens to generate *z*-stacks of images covering the dendritic extent of neurons (1 μm *z*-steps, 2,048^2^ pixel resolution) to allow identification of somatodendritic arborizations and to facilitate neuronal reconstruction. Sections of secondary or tertiary dendrite from *str. L-M, radiatum,* and *oriens* were imaged under high magnification (63×; 2,048^2^ resolution; 2× zoom; pixel width, 0.04 µm; *z*-step size, 0.13 µm) to facilitate deconvolution of imaged dendrites (Huygens Deconvolution, Scientific Volume Imaging).

For plaque loading analysis, acute slices were imaged in CA1 at low magnification to identify labeled neurons (using Alexa Fluor 633 signal) and ascertain the density of Aβ plaques (using Alexa Fluor 488 signal). Overview confocal *z*-stacks of images from the slice with respect to recorded cells were taken. Based on these *z*-stack images, the Aβ-positive area was quantified by applying a 4 pixel Gaussian blur to exclude noise and better define plaques, and then images were binarized using the Triangle Algorithm. From binarized images, plaque volume was calculated, as a percentage of the total field of view, and then correlated with the dendritic length of recorded neurons.

CA1 pyramidal neurons were reconstructed offline from the above image stacks using a semiautomatic analysis software (Simple Neurite Tracer plug-in for the ImageJ/Fiji software package; http://fiji.org; Arshadi et al., 2021). Neurons were rejected for reconstruction if dendrites had been cut during slice preparation. Basal dendrites (extending from the soma into str. oriens) and apical dendrites (extending from the primary apical dendrite into *str. radiatum* and *str. L-M*) were reconstructed and analyzed separately. Reconstructions were then analyzed based on Sholl distribution, dendritic length, and branch order analysis.

To confirm synapse loss in APP/PS1 mice through aging, the density of dendritic spines on CA1 pyramidal neuron dendrites was calculated. Prior to spine density analysis, high-magnification images of dendrites in *str. L-M, radiatum,* and *oriens* were deconvolved using Huygens Professional (Scientific Volume Instruments). To avoid experimenter bias, dendritic spines were identified using DeepD3, a deep learning-based framework for dendritic spine segmentation (Fernholz et al., 2024). In brief, a custom DeepD3 model was trained using a set of deconvolved images of dendrites from str. L-M, radiatum, and oriens, from both WT and Het mice, whose spines and dendrite shaft had been labeled pixel-wise using PiPrA (Gómez et al., 2020). The model predicts the presence of putative dendritic spines in the images, which were then thresholded by prediction likelihood, segmented, and counted using a custom Python workflow.

### Statistics

All data were collected blind to genotype. All experimental group sizes were determined a priori by power analysis, based on expected effect sizes from earlier studies on GABA_B_ and Kir3 protein localization in the APP/PS1 mouse (Martín-Belmonte et al., 2020a,b, 2022, 2025) and the variability of GABA_B_R-mediated effects at pre- and postsynaptic compartments (Brown et al., 2007; Booker et al., 2013, 2017, 2018, 2020; Watson and Booker, 2024). All data are presented as either mean ± SEM or median ± 95% confidence interval and displayed as boxplots with individual replicates shown overlain, which are indicated in figure legends. To prevent the risk of Type 1 statistical errors arising from the sampling of multiple cells/measures from the same animal, we subjected all data to linear mixed-effect modeling (or the generalized form of such). These models take into account variability arising from random effects, including animal, slice, and litter while testing the effect size, variability, and statistical significance of fixed effects, including age, GABA_B_R-mediated effects, and neuronal structure. This approach aims to account for differences between biological replicates while appreciating the cell-to-cell variation as a major source of variability.

### Description of outcome-neutral criteria

To confirm that our data represent a biologically meaningful finding, relating to inhibitory receptor function in a mouse model of AD, we have identified a number of key outcome-neutral criteria to assure the validity of our findings. First, as GABA_B_Rs and Kir3 channels have reduced expression in the APP/PS1 mouse, we performed western blot analysis, probing for GABA_B1_, and Kir3.2 from synaptosomes prepared from the acute hippocampus from APP/PS1 and littermate controls, age grouped according to the electrophysiological recordings. These were compared with the nonsynaptosome fraction to determine whether loss of these proteins is cell wide or synaptic localization. Second, we performed immunohistochemistry in the recorded brain slices for Aβ to confirm the presence of plaques in the brain regions tested. Third, performed dendritic spine counts from the recorded cells to confirm that putative excitatory synapses display reduced density. This confirms consistency with other studies in the APP/PS1 mouse where synaptic loss has been reported (Knafo et al., 2009; Alonso-Nanclares et al., 2013; Hong et al., 2016; Oyelami et al., 2016; Viana da Silva et al., 2016). Finally, synapse loss was confirmed by measurement of the number of spontaneous synaptic events. This confirmed that there was a functional loss of excitatory synapses as previously reported (Viana da Silva et al., 2016) and serve as a control for the potential functional GABA_B_R currents observed in the same animals.

### Timeline

It is expected that this project will be completed within 9 months of preregistration acceptance. We intend to perform all aims at three distinct age groups 1, 6, and 12 months of age. These experiments will take advantage of ongoing breeding within the department. All data streams are expected to be generated from the same animals in parallel; as such, once the calculated power of individual experiments has been satisfied, final data analysis, imaging, and preparation of the final manuscript would be expected ∼14 months.

### Data availability plan

All raw and analyzed data generated from this study will be deposited in an online repository (University of Edinburgh DataShare). Analyzed data contributing to final manuscript figures and interpretation will provided as supplementary datasets. All neuronal reconstructions will be uploaded to online databases (e.g., NeuroMorph).

### Ethical approval plan

All experiments will be performed in accordance with UK Home Office (ASPA, 1986) under an existing project license (PCB113BFD and PP8710936). All breeding and maintenance of experimental animals is conducted according to local (Biological Veterinary Services, University of Edinburgh) ethical approvals and guidelines.

## Results

In the present study, we tested the hypothesis that functional GABA_B_R signaling is impaired in the APP/PS1 mouse model of Aβ pathology. We aimed to determine whether GABA_B_R pre- and postsynaptic signaling display age- and genotype-specific differences, which may explain cellular and synaptic vulnerability in AD. For this, we examined APP/PS1 mice at 1, 6, and 12 months of age alongside hippocampal OSCs at 4–6 weeks using whole-cell and LFP electrophysiology, biochemical assays, and cellular neuroanatomy.

### Morphology of CA1 pyramidal neurons in APP/PS1 mice

**Figure 1.**
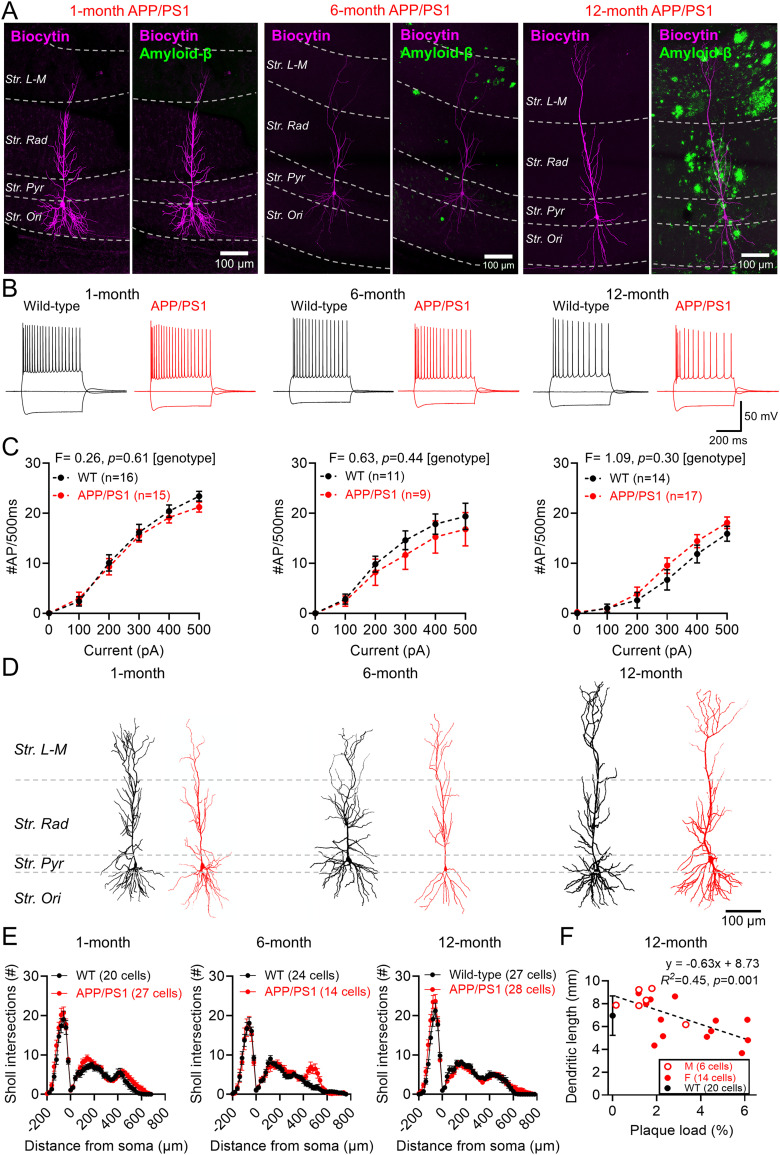
Characterization of CA1 pyramidal cells in ex vivo brain slices from the APP/PS1 mouse across the mouse lifespan. ***A***, Flattened confocal images of CA1 showing a biocytin-filled CA1 pyramidal cell (magenta) and immunolabeling for Aβ (green) with recorded pyramidal cells superimposed for brevity, at 1 (left), 6 (middle), and 12 months (right) of age. ***B***, Voltage responses of CA1 pyramidal cells to −500, 0, and +500 pA from WT (black) and APP/PS1 (red) mice, at the same ages. ***C***, The AP number measured for each current step from all recorded CA1 pyramidal cells at 1 month (WT, 16 cells; 9 mice; APP/PS1, 15 cells; 10 mice), 6 months (WT, 11 cells, 9 mice; APP/PS1, 9 cells, 6 mice), and 12 months (WT, 14 cells, 9 mice; APP/PS1, 17 cells, 13 mice). ***D***, Example somatodendritic reconstructions of CA1 pyramidal cells for WT (black) and APP/PS1 (red) mice at each age. ***E***, Sholl analysis plotted as distance from soma (0 μm) for basal (negative values) and apical (positive values) from 1 month (WT, 20 cells, 9 mice; APP/PS1, 27 cells; 10 mice), 6 months (WT, 24 cells, 10 mice; APP/PS1, 14 cells, 6 mice), and 12 months (WT, 27 cells, 9 mice; APP/PS1, 28 cells, 13 mice). ***F***, Correlation of total dendritic length for CA1 pyramidal cells from 12-month-old APP/PS1 mice with plaque load, expressed as the percentage of the total area. Data are shown for female (filled circles; 14 cells, 9 mice) and male (open circles, 6 cells, 3 mice) APP/PS1 mice, with the WT average ± SD shown for comparison (black; 8 mice). The dashed line reflects the result of linear regression analysis. Data shown as mean ± SEM (***C***, ***E***). Statistics shown for Pearson's analysis (***F***).

First, we confirmed the accumulation of Aβ plaques with age in the APP/PS1 model, consistent with previous studies of this model (Yan et al., 2009). Immunohistochemical labeling for Aβ in fixed brain slices confirmed the proliferation of Aβ plaques from 6 months of age in APP/PS1 mice, which increasingly covered a greater extent of the CA1 region by 12 months (Fig. 1*A*). Whole-cell patch–clamp recordings from CA1 pyramidal cells in wild-type (WT) and APP/PS1 mice confirmed that these cells produced repetitive trains of APs at all ages (Fig. 1*B*), which did not differ in current-frequency response at any age (Fig. 1*C*). Morphological reconstruction of a subset of recorded neurons confirmed CA1 pyramidal cell identity, which appeared to be broadly similar across age and genotype (Fig. 1*D*). Sholl analysis of neurons from APP/PS1 mice revealed minimal morphological differences at 1 month; subtle differences at 6 months, notably in distal dendritic tufts; and no overt difference in distribution at 12 months (Fig. 1*E*). We found a weak but significant correlation between total plaque density (as a proxy for plaque proximity) with total dendritic length, consistent with previously reported Aβ-dependent dendritic degeneration (Šišková et al., 2014; Fig. 1*F*).

### Postsynaptic GABA_B_Rs in CA1 are not reduced in APP/PS1 mice

**Figure 2.**
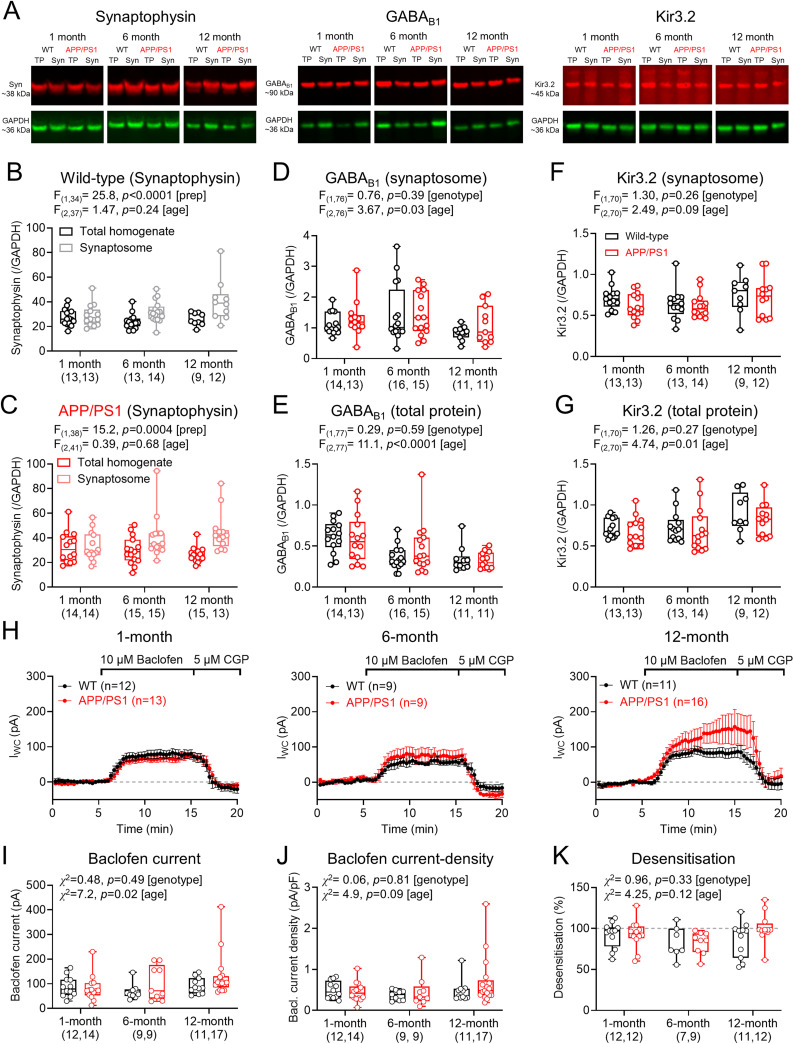
Typical levels of GABA_B_R expression and function in CA1 pyramidal cells of APP/PS1 mice. ***A***, Example immunoblots for synaptophysin, GABA_B1_, and Kir3.2 (red, top) with respect to GAPDH (green, bottom) for 1-, 6-, 12-month-old WT and APP/PS1 mice from total protein (TP) and synaptosome (Syn) fractions. ***B***, Quantification of synaptophysin levels in total protein (black) and synaptosome (gray) fractions from WT mice at 1 (13 mice), 6 (14 mice), and 12 months of age (12 mice), relative to GAPDH. ***C***, Synaptophysin levels in total protein (red) and synaptosome (pink) fractions from APP/PS1 mice at 1 (14 mice), 6 (15 mice), and 12 months (15 mice) of age, relative to GAPDH. ***D***, Quantification of GABA_B1_ labeling in blots prepared from the synaptosomal fraction from WT (black) and APP/PS1 (red) mice (number indicated below graphs), relative to GAPDH. ***E***, Quantification of GABA_B1_ labeling from the total protein fraction from WT (black) and APP/PS1 (red) mice (number indicated below graphs), relative to GAPDH. ***F***, Kir3.2 labeling in synaptosomal blots from WT (black) and APP/PS1 (red) mice (number indicated below graphs), relative to GAPDH. ***G***, Kir3.2 labeling from the total protein fraction from WT (black) and APP/PS1 (red) mice (number indicated below graphs), relative to GAPDH. ***H***, Time-course plots of holding-current responses of CA1 pyramidal cells in response to baclofen (10 μM), then CGP-55,845 (CGP, 5 μM) wash-in, from WT (red) and APP/PS1 (red) mice. Data shown for 1 (left), 6 (middle), and 12 month (right) mice. ***I***, Absolute baclofen current amplitude measure for all genotypes at 1 (WT, 12 cells 9 mice; APP/PS1, 14 cells, 10 mice), 6 (WT, 9 cells, 8 mice; APP/PS1, 9 cells, 6 mice), and 12 months (WT, 11 cells, 9 mice; APP/PS1, 17 cells, 13 mice). ***J***, Baclofen-mediated current–density plotted for all genotypes and ages. ***K***, Desensitization ratio of baclofen-mediated currents plotted for all genotypes and ages; from the same cells at ***I***. Note only 7 cells from 6 mice for 6-month WT and 12 cells from 11 mice for 12-month APP/PS1 were recorded. Data are shown as mean ± SEM (***H***) or box plots, depicting the median with 25–75% quartile range and maximum and minimum (***B–G***, ***I–L***). Data from individual cells (***H–L***) or animals (***B–G***) are shown overlaid.

Prior electron microscopy analysis has reported an age-dependent reduction in dendritic GABA_B_Rs and Kir3 channel expression in CA1 pyramidal cells of the APP/PS1 mouse (Martín-Belmonte et al., 2020a,b, 2022). To determine whether we could observe this, we first performed biochemical analysis of GABA_B1_ and Kir3.2 subunit expression in the hippocampus of 1-, 6-, and 12-month-old APP/PS1 mice and WT littermates from total homogenate and purified synaptosome fractions (Fig. 2*A*) to isolate perisynaptic receptors and channels. First, we confirmed enrichment of the presynaptic protein synaptophysin in the synaptosome fractions from WT (Fig. 2*B*) and APP/PS1 mice (Fig. 2*C*). To determine if GABA_B_Rs displayed genotype-dependent loss at synaptic domains, we probed blots of the synaptosome fraction with antibodies raised against the constitutive GABA_B1_ isoform. We found no difference in GABA_B1_ abundance in the synaptic fraction of APP/PS1 mice compared with WT at any age tested (Fig. 2*D*). In the total homogenate fraction, we found an age-dependent loss of GABA_B1_ subunits, but no impact of genotype (Fig. 2*E*). Conversely, we found an age-dependent increase in Kir3.2 protein levels in the synaptosome fraction, but again this was not related to genotype (Fig. 2*F*). We found no genotype effect on the relative abundance of Kir3.2 in the total homogenate fraction, despite age-dependent increases in abundance (Fig. 2*G*). These data indicate that GABA_B_R and effector Kir3 channels were abundant at synapses, but their synaptic levels were largely unaffected in APP/PS1 mice.

To determine whether functional GABA_B_R postsynaptic currents are altered in the APP/PS1 mouse, we performed whole-cell patch–clamp recordings from CA1 pyramidal cells in the presence of ionotropic glutamate and GABA_A_ receptor antagonists (10 μM CNQX, 50 μM DL-AP5, and 10 μM gabazine) and then applied the canonical GABA_B_R agonist *R*-baclofen (10 μM), followed by the selective antagonist CGP-55,845 (CGP, 5 μM). In slices prepared from 1-, 6-, and 12-month-old mice, *R*-baclofen application uniformly led to a large outward current, consistent with the activation of inwardly-rectifying K^+^ channels, which was blocked when CGP was applied to the bath (Fig. 2*H*). Quantification of the peak baclofen current recorded in CA1 pyramidal cells revealed no difference between APP/PS1 mice compared with WT at any age tested (Fig. 2*I*). As we observed some differences in dendritic length, particularly associated with Aβ plaque density, we normalized these peak currents to membrane capacitance—a proxy for cell size (Degro et al., 2024). Baclofen current-density analysis revealed no genotype differences, irrespective of age (Fig. 2*J*).

Native GABA_B_Rs interact with many proteins, including APP (Dinamarca et al., 2019) and auxiliary KCTD proteins, the latter of which regulate desensitization of endogenous postsynaptic GABA_B_R currents (Schwenk et al., 2010; Fritzius et al., 2017). Therefore, we examined if desensitization of baclofen currents was dysregulated in the APP/PS1 mice. We found minimal desensitization of baclofen currents in CA1 pyramidal cells in WT mice and found no significant differences with APP/PS1 mice at any age (Fig. 2*K*).

Together, these data indicate that functional GABA_B_Rs are expressed at high levels on the dendrites of CA1 pyramidal cells throughout adult life, and this does not differ in APP/PS1 mice.

### Reorganization of GABA_B_R signaling in apical dendrites of APP/PS1 mice, independent of age

**Figure 3.**
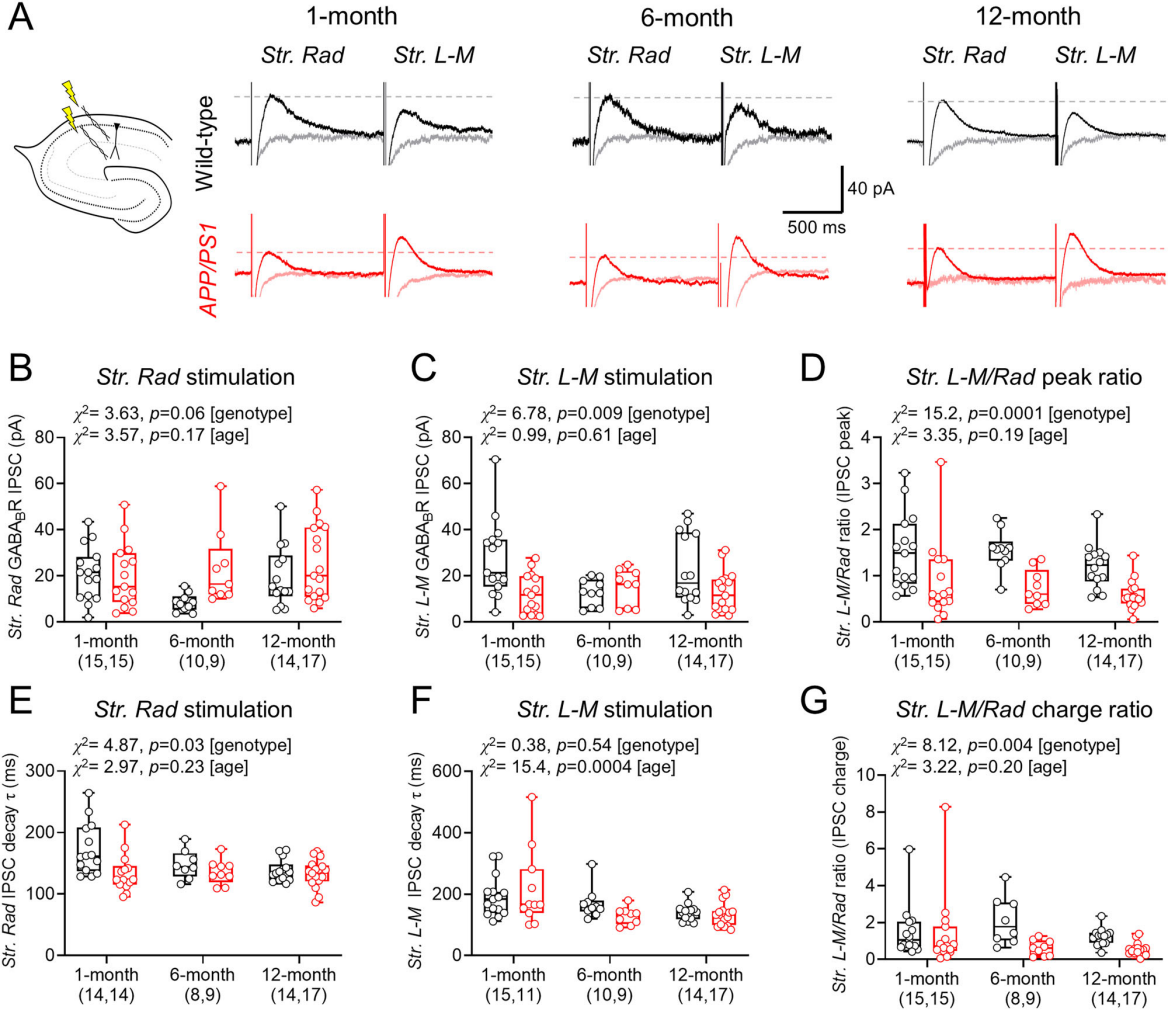
Loss of GABA_B_R-mediated IPSCs from CA1 pyramidal cell distal dendrites in APP/PS1 mice is independent of age. ***A***, Left, Schematic of recording configuration, indicating dual-site stimulation. Right, Example GABA_B_R-mediated IPSCs elicited by str. radiatum (str. radiatum) and str. lacunosum-moleculare (str. L-M) from WT (black, top traces) and APP/PS1 mice (red, bottom). Peak amplitude of str. radiatum IPSCs are indicated (dashed lines) as well as the same responses in the presence of CGP (gray, pink). ***B***, GABA_B_R-mediated IPSC peak amplitudes from WT and APP/PS1 mice, in response to str. radiatum stimulation; at 1 (WT, 15 cells, 9 mice; APP/PS1, 15 cells, 10 mice), 6 (WT, 10 cells, 8 mice; APP/PS1, 9 cells, 6 mice), and 12 months (WT, 14 cells, 9 mice; APP/PS1, 17 cells, 13 mice). ***C***, Str. L-M stimulated GABA_B_R-mediated IPSC amplitudes from WT and APP/PS1 mice. ***D***, Rationalized str. L-M GABA_B_R-mediated IPSC amplitudes to str. radiatum IPSCs. ***E***, Measured monoexponential decay-time constant of str. radiatum-evoked GABA_B_R–mediated IPSCs. ***F***, Decay-time constants of GABA_B_R-mediated IPSCs in str. L-M. ***G***, Rationalized str. L-M to str. radiatum GABA_B_R-mediated IPSC charge transfer. Note slightly lower cell numbers for kinetics and charge transfer. Data are shown as box plots depicting the median with 25–75% quartile range and maximum and minimum. Data from individual cells are shown overlaid.

GABA_B_R subunits have been suggested to display layer-specific loss in APP/PS1 mice (Martín-Belmonte et al., 2020a,b, 2022); thus, we next performed experiments using dual-site stimulation to evoke slow IPSCs in* str. radiatum* and *str. L-M* of the CA1. IPSCs were elicited in pyramidal cells following 5× 200 Hz stimuli (50 V stimulus strength, 20 s intervals) at −65 mV voltage clamp in the presence of 10 μM CNQX, 50 μM DL-AP5, and 10 μM gabazine using a K-gluconate–based internal solution. The resulting evoked slow-IPSCs were fully blocked by CGP application, confirming that they are GABA_B_R dependent (Fig. 3*A*). Measurement of GABA_B_R-mediated IPSC amplitudes following *str. radiatum* stimulation revealed higher response amplitudes in APP/PS1 mice compared with WT, but with no effect of age (Fig. 3*B*). Conversely, GABA_B_R-mediated IPSCs resulting from* str. L-M* stimulation were smaller in APP/PS1 mice (Fig. 3*C*). As there was high cell-to-cell variability in response amplitudes, we compared the amplitude of* str. radiatum* and *str. L-M* GABA_B_R-mediated IPSC amplitudes within each cell. WT mice consistently had larger GABA_B_R-mediated IPSCs in *str. L-M* relative to *str. radiatum*. In contrast, APP/PS1 mice had lower GABA_B_R-mediated IPSCs in* str. L-M* relative to *str. radiatum*, irrespective of age (Fig. 3*D*).

As the total inhibition mediated by GABA_B_Rs depends on the duration of IPSCs (Watson and Booker, 2024), we next asked if the kinetics of GABA_B_R-mediated IPSCs differed between genotypes. Measurement of the decay-time constants of IPSCs revealed faster decays in APP/PS1 mice, relative to WT, following *str. radiatum* stimulation (Fig. 3*E*). This was not observed following *str. L-M* stimulation, despite an age-dependent decrease in decay times (Fig. 3*F*). This difference in decay times did not account for genotype effects on GABA_B_R-mediated inhibition, with* str. L-M/str. radiatum* IPSC charge transfer ratio being lower in APP/PS1, irrespective of age (Fig. 3*G*). These findings confirm that functional GABA_B_R-mediated currents display layer-specific differences in CA1 pyramidal cells of APP/PS1 mice, but these appear unrelated to age.

### GABA_B_R-dependent inhibition of glutamatergic, but not GABAergic, presynaptic release is impaired in APP/PS1 mice

**Figure 4.**
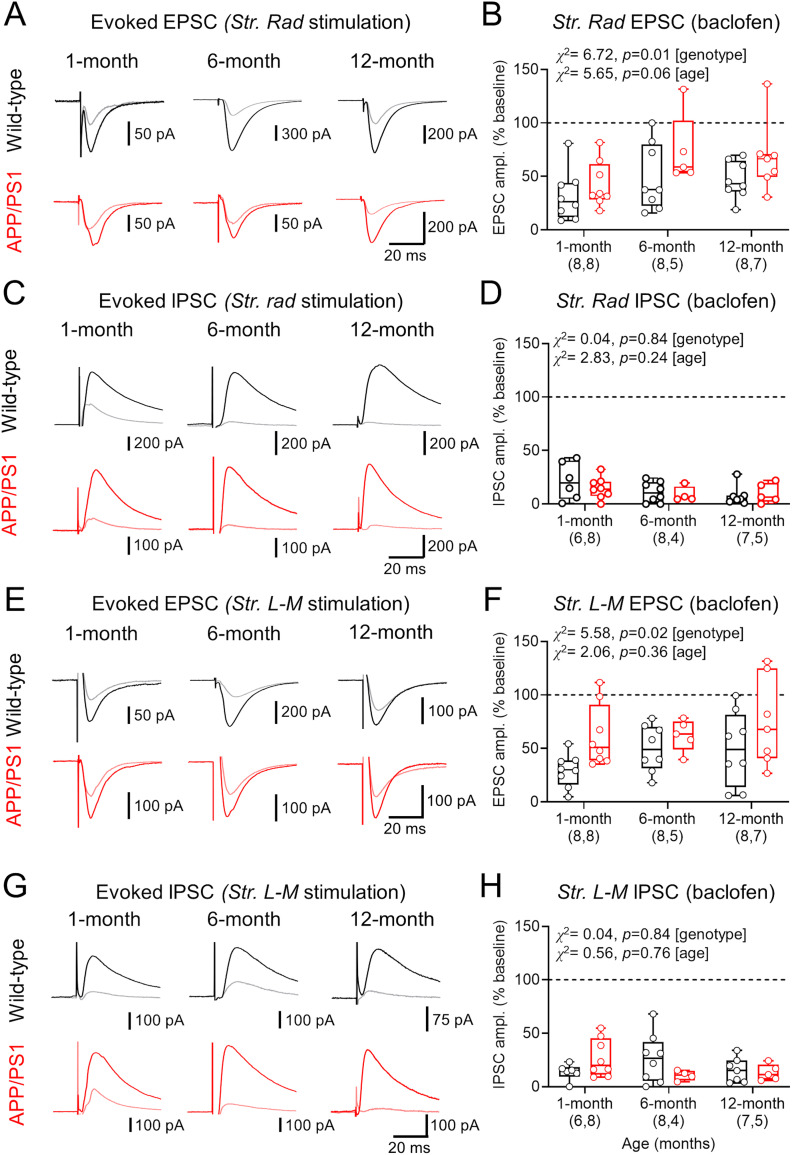
Impaired presynaptic GABA_B_R-mediated inhibition of inputs to CA1 pyramidal cells in APP/PS1 mice. ***A***, Example EPSCs resulting from stimulation of *str. radiatum* for WT (black, top) and APP/PS1 (red, bottom). EPSCs evoked in the presence of 10 μM *R-*baclofen are shown (WT, gray; APP/PS1, pink), for all ages tested. ***B***, The change in EPSC amplitude measured (as the percentage of baseline EPSC amplitudes) following 5 min *R-*baclofen application for WT and APP/PS1 pyramidal cells. ***C***, Example *str. radiatum* IPSCs in CA1 pyramidal cells, according to the same scheme as ***A***. ***D***, Change in *str. radiatum* IPSC amplitude (compared with baseline) following baclofen wash-in. ***E***, Example EPSCs recorded in CA1 pyramidal cells following *str. L-M* stimulation, according to the same scheme as ***A***. ***F***, *Str. L-M* EPSC amplitude change (compared with baseline) following *R-*baclofen wash-in. ***G***, Example *str. L-M* IPSCs. ***H***, *Str. L-M* IPSC amplitude change (compared with baseline) following *R-*baclofen wash-in. Data are shown from 1 (WT, 7 mice; APP/PS1, 8 mice), 6 (WT, 7 mice; APP/PS1, 4 mice), and 12 months (WT, 6 mice; APP/PS1, 8 mice) with the number of cells indicated on graphs, with box plots depicting the median with 25–75% quartile range, and maximum and minimum. Data from individual cells are shown overlaid.

Presynaptic GABA_B_R function has been previously shown to be modified by the expression of APP (Dinamarca et al., 2019), and presynaptic receptor subunits are indicated as lower in electron microscopy studies (Martín-Belmonte et al., 2020a,b, 2022). To systematically determine the degree of GABA_B_R presynaptic inhibition in APP/PS1 mice in a synapse-specific manner, we performed whole-cell patch–clamp recordings of EPSCs (−70 mV) or IPSCs (0 mV) from CA1 pyramidal cells. Synaptic responses were evoked via bipolar stimulating electrodes placed in *str. radiatum* and *str. L-M*, with EPSC amplitudes titrated to give large, monosynaptic EPSCs (typically 100–200 pA). Bath-applied 10 μM *R*-baclofen was used to determine the influence of presynaptic GABA_B_Rs on synaptic transmission. Monosynaptic EPSCs were evoked in *str. radiatum*, which were consistently inhibited by baclofen application at all ages tested, in both genotypes (Fig. 4*A*). Quantification of baclofen-mediated inhibition of *str. radiatum*-evoked EPSCs revealed that APP/PS1 mice consistently showed less inhibition, with both genotypes displaying age-dependent reductions in inhibition (Fig. 4*B*). For IPSCs evoked by* str. radiatum* stimulation, baclofen strongly attenuated responses (Fig. 4*C*), which did not differ between genotypes (Fig. 4*D*). *Str. L-M* evoked EPSCs also showed moderate GABA_B_R-dependent inhibition (Fig. 4*E*), with APP/PS1 mice again displaying reduced baclofen sensitivity, albeit with no effect of age in either genotype (Fig. 4*F*). We observed very strong inhibition of *str. L-M* IPSCs (Fig. 4*G*), which did not differ between genotypes (Fig. 4*H*). Together, these data confirm that in APP/PS1 mice, presynaptic GABA_B_R inhibition is reduced over the entire lifespan but specific to glutamatergic inputs.

### Presynaptic input strength to CA1 pyramidal cells in the APP/PS1 mouse is reduced but are equally sensitive to GABA_B_R activation compared with WTs

**Figure 5.**
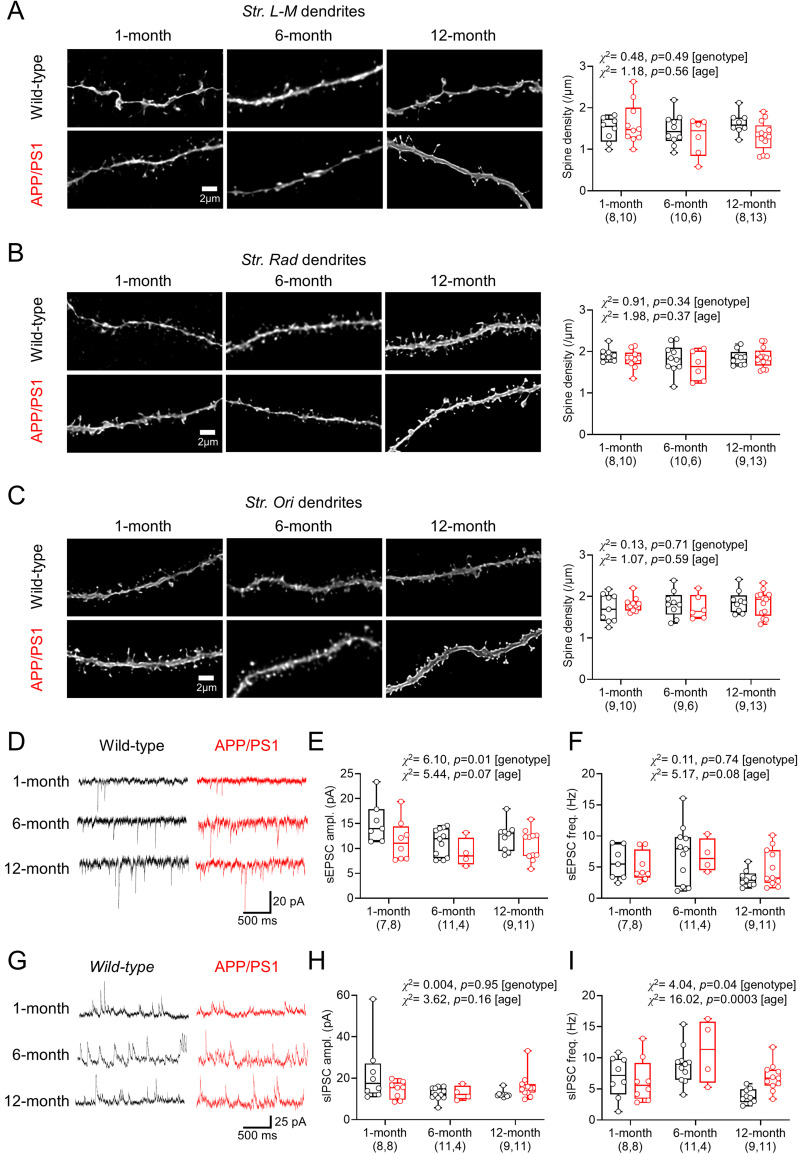
No change in synaptic density but impaired spontaneous synaptic communication in CA1 pyramidal cells from APP/PS1 mice. ***A***, Right, Example deconvolved dendritic segments from *str. L-M* dendrites from biocytin-filled CA1 pyramidal cells from WT (top) and APP/PS1 (bottom) mice, at all ages tested. Left, Dendritic spine density plotted for dendrites in *str. L-M* from WT (black) and APP/PS1 (red) CA1 pyramidal cells. ***B***, The same data, but for *str. radiatum* dendrites. ***C***, The same data, but for *str. oriens* dendrites. Dendritic spine data shown from 1 (WT, 9 mice; APP/PS1, 10 mice), 6 (WT, 10 mice; APP/PS1, 6 mice), and 12 months (WT, 9 mice; APP/PS1, 13 mice) as indicated on graphs, with animal average data plotted. Example recordings of spontaneous (s)EPSCs recorded from WT (black) and APP/PS1 (red) CA1 pyramidal cells at −70 mV. Comparison of the sEPSC amplitude (***E***) and frequency (***F***), from both genotypes, at all ages. ***G***, Example sIPSC recordings from WT (black) and APP/PS1 (red) CA1 pyramidal cells at 0 mV. Comparison of the sIPSC amplitude (***H***) and frequency (***I***) from both genotypes at all ages. sEPSC/IPSC data shown from 1 (WT, 7 mice; APP/PS1, 8 mice), 6 (WT, 10 mice; APP/PS1, 4 mice), and 12 months (WT, 7 mice; APP/PS1, 10 mice), with the number of cells indicated on graphs. Data are shown as box plots depicting the median with 25–75% quartile range and maximum and minimum. Data from individual cells are shown overlaid.

To determine whether basal changes in spine density (Viana da Silva et al., 2016) may account for presynaptic dysfunction, we next performed high-resolution imaging of dendritic spines on biocytin-filled cells, imaging distal dendrites in *str. L-M* (Fig. 5*A*), proximal oblique dendrites in *str. radiatum* (Fig. 5*B*), and basal dendrites in* str. oriens* (Fig. 5*C*). To measure spine density in an unbiased manner, we identified the number of putative dendritic spines using a machine-learning–based approach (DeepD3; Fernholz et al., 2024). We observed no genotype-dependent differences in spine density at any age tested (Fig. 5*A–C*). We next functionally validated these findings by measuring sEPSCs (Fig. 5*D*). APP/PS1 mice had smaller amplitude sEPSCs than WT littermates (Fig. 5*E*). Consistent with no changes in spine density, we found no genotype-dependent changes in sEPSC frequency (Fig. 5*F*). Measuring sIPSCs (Fig. 5*G*) from the same CA1 pyramidal cells, held at 0 mV instead, showed that amplitude (Fig. 5*H*) did not display genotype-specific differences. However, sIPSC frequency was higher in APP/PS1 mice, which also displayed prominent age-dependent variation (Fig. 5*I*).

**Figure 6.**
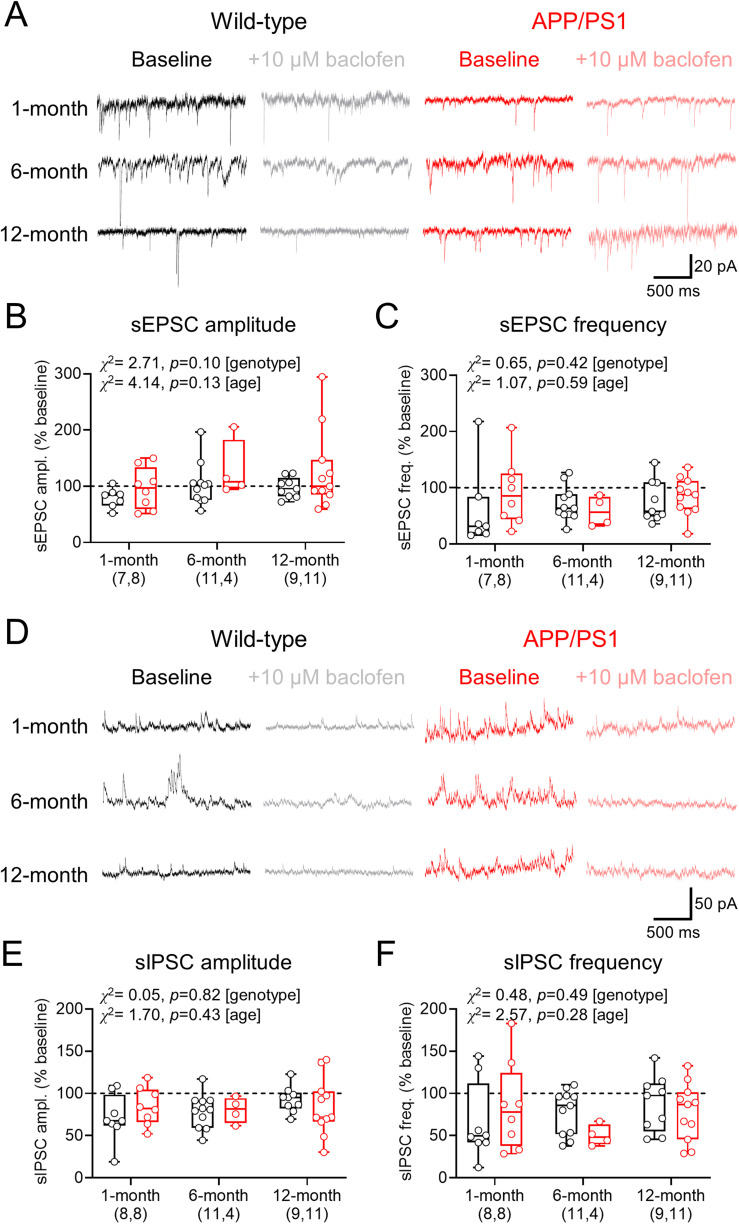
No overt effect of GABA_B_Rs on sEPSCs and sIPSCs in CA1 pyramidal cells from APP/PS1 mice. ***A***, Example sEPSCs recorded from CA1 pyramidal cells at −70 mV from WT (black) and APP/PS1 (red) mice, before and after 10 μM *R-*baclofen application (gray and pink, respectively), for all ages tested. ***B***, Change in sEPSC amplitude, measured as the change following baclofen bath application compared with baseline. ***C***, Change in sEPSC frequency relative to baseline following baclofen application. ***D***, Example sIPSCs recorded at −0 mV from CA1 pyramidal cells from WT and APP/PS1 mice, before and after 10 μM baclofen application, for all ages tested. ***E***, *R-*baclofen-mediated change in sIPSC amplitude following bath application. ***F***, Change in sIPSC frequency relative to baseline following *R-*baclofen application. Data shown from 1 (WT, 7 mice; APP/PS1, 8 mice), 6 (WT, 10 mice; APP/PS1, 4 mice), and 12 months (WT, 7 mice; APP/PS1, 10 mice), with the number of cells indicated on graphs and plotted as box plots depicting the median with 25–75% quartile range, and maximum and minimum. Data from individual cells are shown overlaid.

To determine whether GABA_B_R activation affected spontaneous synaptic release, we next applied 10 μM *R-*baclofen to the bath and assessed sEPSCs (Fig. 6*A*). Quantification revealed that baclofen-mediated inhibition of sEPSC amplitude did not display genotype-specific differences (Fig. 6*B*) nor did sEPSC frequency (Fig. 6*C*). sIPSC recordings performed at 0 mV confirmed that baclofen application attenuated inhibitory synaptic function (Fig. 6*D*), which did not differ between WT and APP/PS1 mice in either amplitude (Fig. 6*E*) or frequency (Fig. 6*F*).

Taken together, these data confirm that there are minimal changes in dendritic spine density in the APP/PS1 mouse, even when Aβ plaque density is high. We find that functional changes in glutamatergic and GABAergic synaptic transmission are present under baseline conditions but are not differentially inhibited by GABA_B_R activation.

### Altered sensitivity of gamma oscillations to GABA_B_R activation in adult APP/PS1 mice

**Figure 7.**
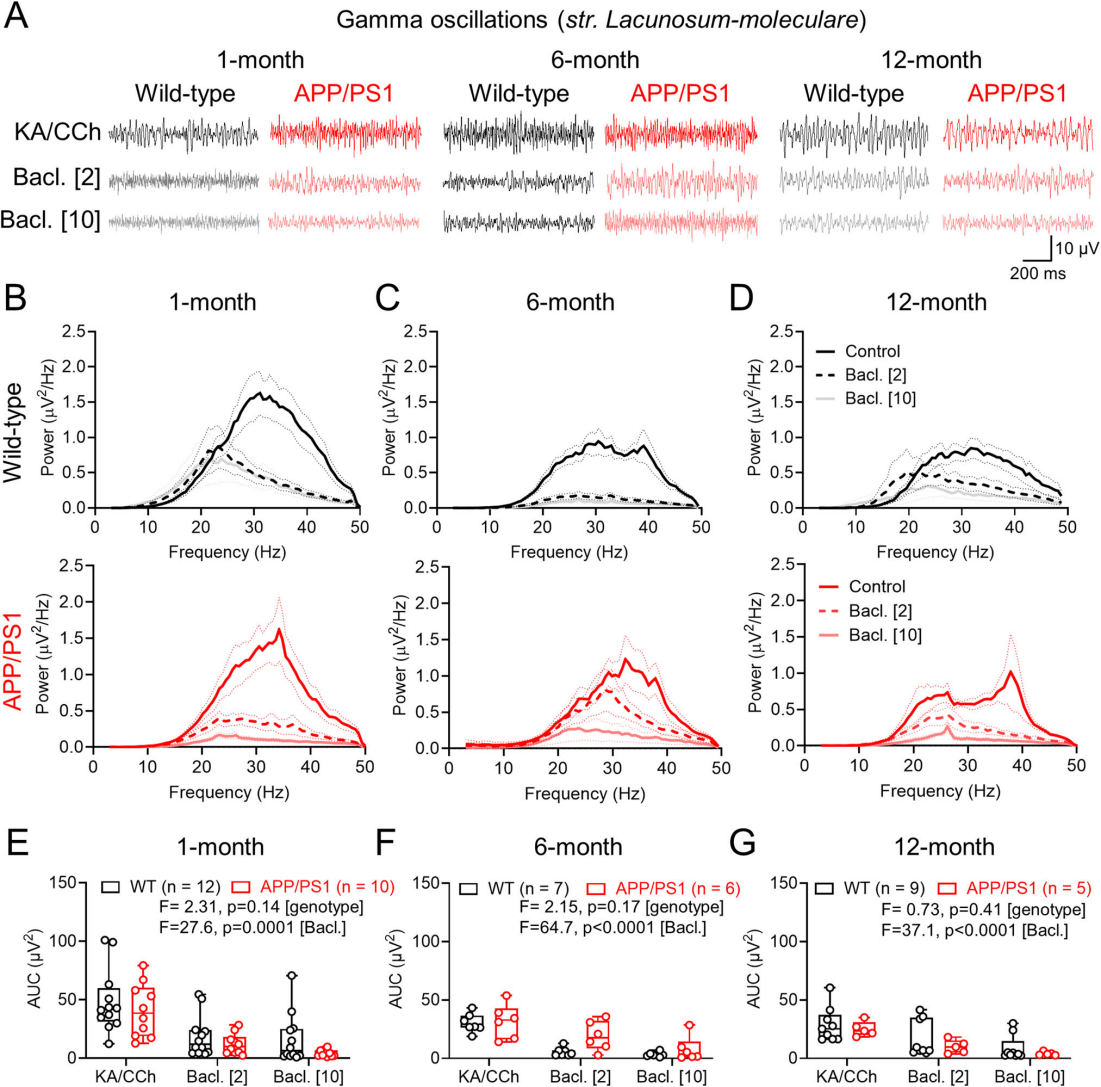
Gamma oscillations in *str. L-M* of CA1 are unchanged in APP/PS1 mice in relation to strength and baclofen sensitivity. ***A***, Example LFP recordings from *str. L-M* from WT (black) and APP/PS1 (red) mice, at all ages tested and under control conditions (top), or following wash-in of 2 μM (middle) or 10 μM (bottom) *R-*baclofen (Bacl.). ***B***, Power spectra of WT (black, top) and APP/PS1 (red, bottom) at 1 month of age, in the presence of kainate (KA; 50 nM) and carbachol (CCh; 2.5 μM) then following 2 μM (dark gray/pink) or 10 μM (light gray/pink) *R-*baclofen. ***C***, The same data, but for 6-month-old mice. ***D***, The same data, but for 12-month-old-mice. ***E***, Measured AUC for each genotype at 1-month in KA/CCh alone, or following 2 and 10 μM baclofen. ***F***, The same data but for 6-month-old mice. ***G***, The same data but for 12-month-old mice. Data shown from 1 (WT, 12 mice; APP/PS1, 10 mice), 6 (WT, 7 mice; APP/PS1, 6 mice), and 12 months (WT, 9 mice; APP/PS1, 5 mice), with the number of slices indicated on graphs. Data are shown as mean ± SEM (***B*–*D***) or box plots, depicting the median with 25–75% quartile range, and maximum and minimum (***E–G***). Data from individual slices are shown overlaid.

**Figure 8.**
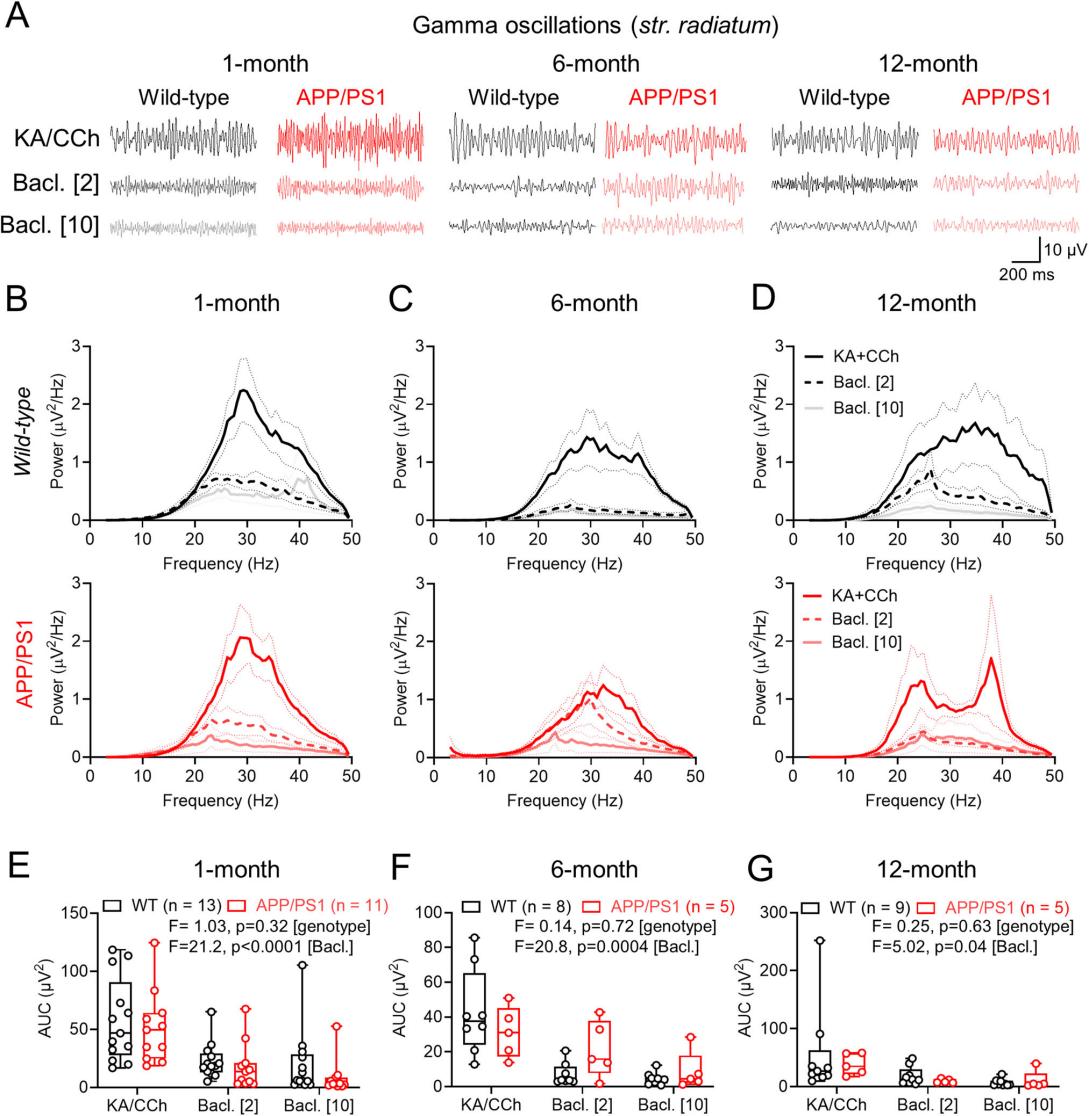
Gamma oscillations in *str. radiatum* of CA1 are typical in APP/PS1 mice in relation to strength and *R-*baclofen sensitivity. ***A***, Example LFP recordings from str. radiatum from WT (black) and APP/PS1 (red) mice, at all ages tested and under control conditions (top), or following wash-in of 2 μM (middle) or 10 μM (bottom) *R-*baclofen (Bacl.). ***B***, Power spectra of WT (black, top) and APP/PS1 (red, bottom) at 1 month of age, in the presence of kainate (KA, 50 nM) and carbachol (CCh, 2.5 μM) then following 2 μM (dark gray/pink) or 10 μM (light gray/pink) baclofen. ***C***, The same data, but for 6-month-old mice. ***D***, The same data, but for 12-month-old-mice. ***E***, Measured AUC for each genotype at 1 month in KA/CCh alone or following 2 and 10 μM baclofen. ***F***, The same data but for 6-month-old mice. ***G***, The same data but for 12-month-old mice. Data shown from 1 (WT, 13 mice; APP/PS1, 11 mice), 6 (WT 8 mice; APP/PS1, 5 mice), and 12 months (WT, 9 mice; APP/PS1, 5 mice), with the number of slices indicated on graphs. Data are shown as mean ± SEM (***B–D***) or box plots, depicting the median with 25–75% quartile range, and maximum and minimum (***E–G***). Data from individual slices are shown overlaid.

So far, we have shown that postsynaptic GABA_B_R signaling is largely preserved, albeit with laminar reorganization, and that presynaptic inhibition of glutamatergic inputs is weaker in the CA1 of APP/PS1 mice. To determine what effect this has on circuit function, we next performed LFP recordings from *str. L-M* (Fig. 7) and *str. radiatum* (Fig. 8), in which we induced gamma (30–100 Hz) oscillations in liquid/gas interface recordings through bath application of kainate (50 nM) and carbachol (2.5 μM). Following induction of gamma oscillations, we then bath applied presynaptic selective concentrations of baclofen (2 μM; Booker et al., 2020) and then nonselective concentrations (10 μM) to examine the differential effects of GABA_B_Rs.

Analysis of *str. L-M* LFP recordings revealed that baclofen dose-dependently decreased gamma band oscillations in both WT and APP/PS1 mice at 1-, 6-, and 12-months of age (Fig. 7*A*). Gamma power, irrespective of genotype and age, was highly sensitive to baclofen application over the entire frequency range (Fig. 7*B–D*). Analysis of *str. L-M* oscillations revealed a main effect of baclofen but no effect of genotype in both 1-month-old (Fig. 7*E*), 6-month-old (Fig. 7*F*), or 12-month-old mice (Fig. 7*G*). Similarly, we found no evidence for a baclofen × genotype interaction in any age group. Similar data generated in *str. radiatum* revealed no genotype differences of baclofen sensitivity on gamma oscillations (Fig. 8). These data confirm that divergent GABA_B_R signaling may contribute to altered circuit function, but this is likely mediated by differential presynaptic effects and not through altered postsynaptic receptor function.

### Divergent cellular function but normal GABA_B_R signaling in OSCs of APP/PS1 mice

**Figure 9.**
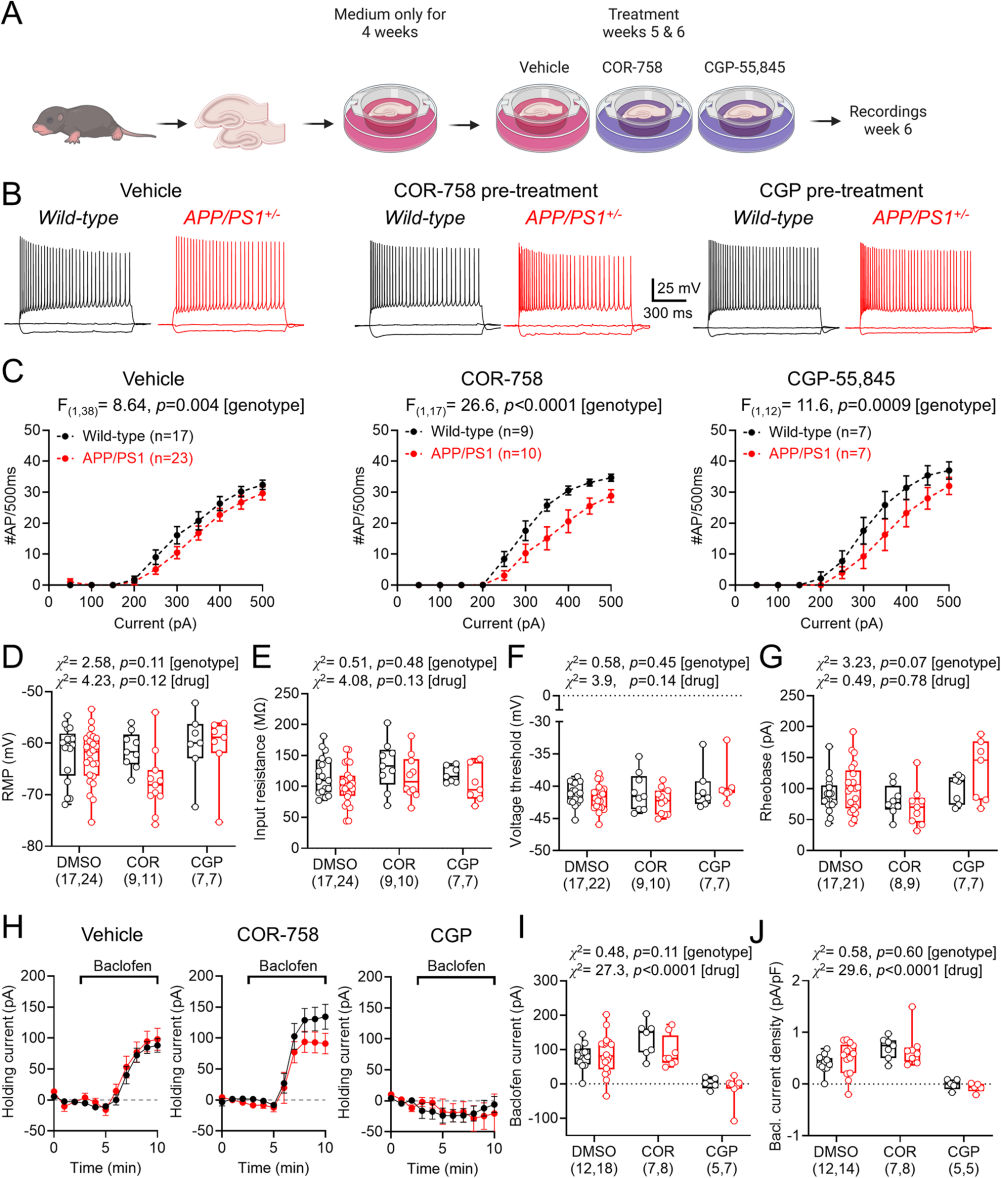
The absence of GABA_B_R deficits in OSCs from APP/PS1 mice, which are not affected by receptor modulation. ***A***, Schematic of organotypic slice preparation, treatment, and recording timeline. ***B***, Example voltage responses of CA1 pyramidal cells from WT (black) and APP/PS1 (red) following depolarizing current injections in cultures treated with vehicle (DMSO, left), COR-758 (20 μM, middle), or CGP (5 μM, right) for 2 weeks. ***C***, Current-frequency response of CA1 pyramidal cells in OSCs prepared from WT (black) and APP/PS1 (red) mice, for vehicle, COR-758, and CGP treatments and recorded at ∼6 weeks in vitro. ***D***, Resting membrane potentials recorded in CA1 pyramidal cells from vehicle, COR-758 (COR), and CGP-treated OSCs from WT and APP/PS1 mice. Comparison of input resistance (***E***), voltage-threshold (***F***), and rheobase (***G***) for the same groups. ***H***, Time-course plots of baclofen-mediated currents in CA1 pyramidal cells held at −65 mV voltage-clamp from vehicle (DMSO), COR-758, and CGP-treated OSCs from WT and APP/PS1 mice. ***I***, Measurement of peak baclofen-mediated currents in WT and APP/PS1 CA1 pyramidal cells for all OSC conditions. ***J***, Baclofen current density for CA1 pyramidal cells from WT and APP/PS1 OSCs, for all conditions. Data shown from cultures prepared from WT (*n* = 12 mice) and APP/PS1 (*n* = 15 mice) mice, with the number of cells indicated on graphs. Data are shown as mean ± SEM (***C***, ***H***) or box plots, depicting the median with 25–75% quartile range, and maximum and minimum (***D–G***, ***I***, ***J***). Data from individual cells are shown overlaid.

It has been previously shown that OSCs prepared from mouse models of amyloid pathology develop pathological features on an accelerated timescale compared with in vivo (Harwell and Coleman, 2016). We next determined whether OSCs prepared from APP/PS1 mice showed similar features to our adult ex vivo assays and if they differentially respond to sustained modulation of GABA_B_R function. For this, we generated OSCs from 6 to 9 d old WT and APP/PS1 mice, which were maintained in vitro for 4–6 weeks. Slices were then treated with either vehicle (DMSO), COR-758 (20 μM, a GABA_B_R NAM), or CGP-55,845 (CGP, 5 μM, a potent and selective antagonist) for a week, following which whole-cell recordings were performed (Fig. 9*A*). Baseline intrinsic physiological recordings from CA1 pyramidal cells in OSCs (Fig. 9*B*) revealed a lower AP discharge in APP/PS1 mice, which was similarly observed after COR-758 or CGP treatment (Fig. 9*C*). Measurement of key electrophysiological properties of CA1 pyramidal cells in OSCs revealed comparable membrane potentials in APP/PS1 mice, irrespective of COR or CGP treatment (Fig. 9*D*). Accordingly, we observed that input resistance (Fig. 9*E*), AP threshold (Fig. 9*F*), and rheobase current (Fig. 9*G*) were all unchanged in CA1 pyramidal cells of APP/PS1 mouse OSCs independent of GABA_B_R inhibition. Bath application of 10 μM *R-*baclofen, when measuring holding current from −70 mV in CA1 pyramidal cells in OSCs, led to large outward whole-cell currents in WT and APP/PS1 mice when treated with vehicle or COR-758, but not when treated with CGP (Fig. 9*H*). Quantification of peak baclofen-mediated current in CA1 pyramidal cells from OSCs confirmed no genotype-specific differences. However, we observed that baclofen-sensitive currents in CA1 pyramidal cells from CGP-treated OSCs were smaller than DMSO controls, while COR-758–treated currents tended to be higher than vehicle (Fig. 9*I*). When normalized to input resistance, the same results were seen (Fig. 9*J*). These data suggest that GABA_B_R signaling in OSCs prepared from APP/PS1 mice is largely preserved, despite subtle baseline differences in excitability. Furthermore, we show that extended application of the antagonist CGP likely leads to reduced GABA_B_R currents, while application of COR-758 does not show this effect.

**Figure 10.**
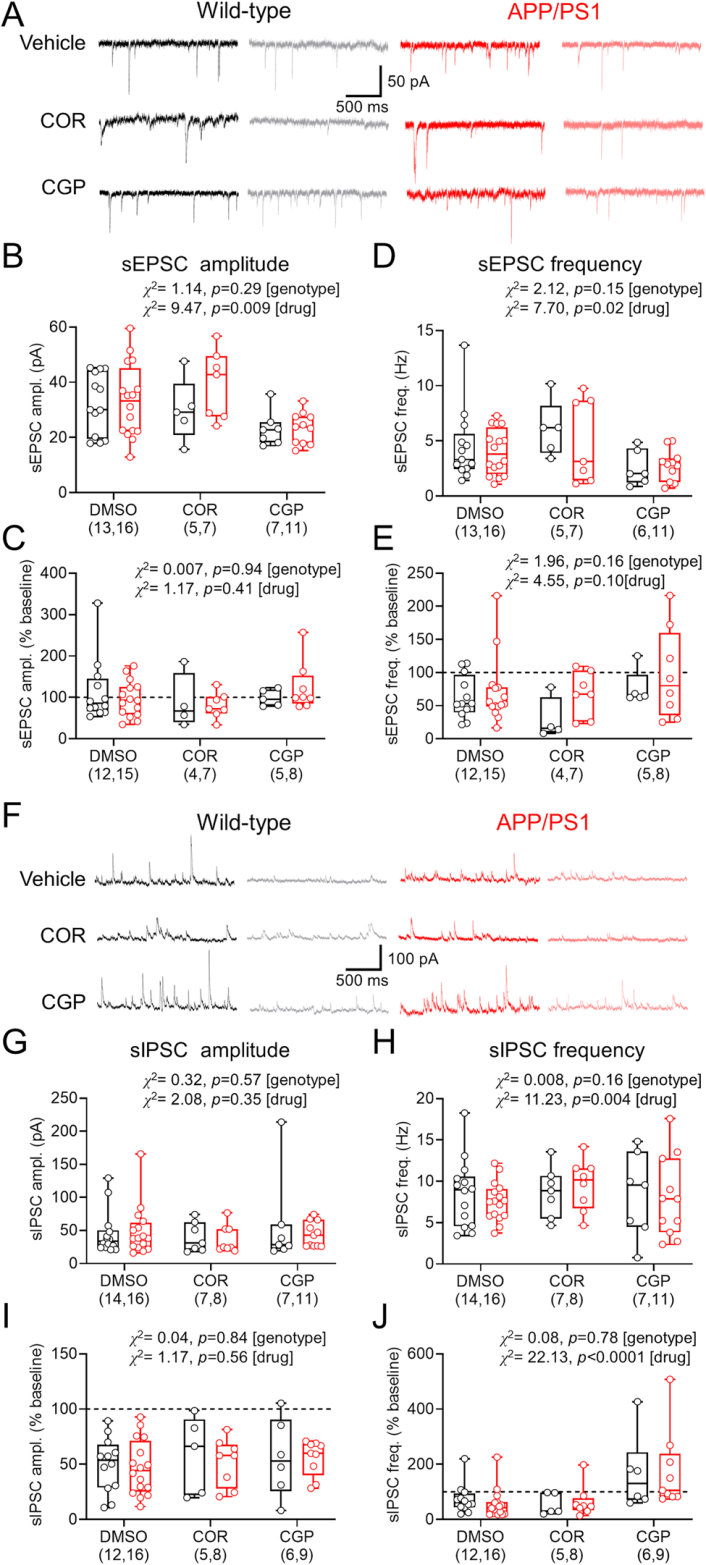
Spontaneous synaptic inputs to CA1 pyramidal cells in APP/PS1 mice are not altered in OSCs and have similar baclofen sensitivity. ***A***, Example sEPSCs recorded from CA1 pyramidal cells in OSCs at −70 mV from WT (black) and APP/PS1 (red) mice, before and after 10 μM baclofen application (gray and pink, respectively), for COR-758 and CGP treatment groups. Comparison of basal sEPSC amplitude (***B***) and relative change (% of baseline) following baclofen wash-in (***C***) from both genotypes and all treatment groups. Comparison of sEPSC frequency (***D***) and relative change (% of baseline) following baclofen wash-in (***E***) from both genotypes and all treatment groups. ***F***, Example sIPSCs recorded from CA1 pyramidal cells in OSCs at −0 mV from WT and APP/PS1 mice, before and after 10 μM baclofen application (gray and pink, respectively), for COR-758 and CGP treatment groups. Comparison of basal sIPSC amplitude (***G***) and relative change (percentage of baseline) following baclofen wash-in (***H***) from both genotypes and all treatment groups. Comparison of sIPSC frequency (***I***) and relative change (% of baseline) following baclofen wash-in (***J***) from both genotypes and all treatment groups. Data shown from cultures prepared from WT (*n* = 12 mice) and APP/PS1 (*n* = 15 mice) mice, with the number of cells indicated on graphs, with box plots depicting the median with 25–75% quartile range, and maximum and minimum. Data from individual cells are shown overlaid.

Finally, we determined if altered basal synaptic transmission was present in APP/PS1 OSCs and whether there was differential sensitivity to presynaptic GABA_B_R activation. For this we recorded sEPSCs (Fig. 10*A*) and sIPSCs (Fig. 10*F*) from CA1 pyramidal cells in OSCs from WT and APP/PS1 mice, treated with vehicle (DMSO), COR-758, or CGP. All recordings were performed with Cs-gluconate internal solution from either −70 mV (sEPSC) or 0 mV (sIPSC). Under control conditions, we found that sEPSC amplitude did not differ between WT and APP/PS1 mice for either vehicle, COR-758, or CGP conditions (Fig. 10*B*), with no difference in baclofen sensitivity either (Fig. 10*C*). Similarly, we found that sEPSC frequency was not altered by genotype, although drug treatment did alter frequency (Fig. 10*D*). We found no genotype or treatment effects of baclofen-mediated inhibition on sEPSC frequency (Fig. 10*E*). Thus, unlike in adult ex vivo brain slices, sEPSCs in OSCs from APP/PS1 mice display WT level synaptic function and GABA_B_R inhibition. For sIPSCs (Fig. 10*F*), we found no genotype or treatment effects on baseline amplitude (Fig. 10*G*) but with a treatment effect on frequency (Fig. 10*H*). We found no baclofen-mediated differences between genotypes or treatment on sIPSC amplitude (Fig. 10*I*). However, despite no genotype effects, CGP pretreatment dramatically prevented baclofen-mediated inhibition of IPSC frequency, independent of genotype (Fig. 10*J*). These data confirm an absence of APP/PS1-dependent modulation of GABAergic synaptic function but reveal that CGP preapplication appears to abolish presynaptic inhibition of inhibition.

## Discussion

In this study, we set out to determine whether functional GABA_B_R signaling was reduced in the APP/PS1 mouse model of amyloidopathy at different stages of Aβ pathology, in line with a number of recent studies in the same model (Martín-Belmonte et al., 2020a,b, 2022). We show that in APP/PS1 mice, GABA_B_R current density is unaltered in the CA1 of the hippocampus at 1, 6, or 12 months of age. While total postsynaptic GABA_B_R currents are unaffected, we observe layer-specific modulation of GABA_B_R-mediated slow IPSCs, independent of age in APP/PS1 mice. Presynaptic GABA_B_R-mediated inhibition of excitatory synaptic transmission was impaired in APP/PS1 mice, but this was also not dependent on age. We found that GABA_B_Rs display similar control of gamma-frequency oscillations in both *str. radiatum* and *str. L-M* at all ages tested. Finally, we show that typical GABA_B_R signaling is preserved in OSCs of APP/PS1 mice, which are equivalently sensitive to pharmacological manipulation of GABA_B_Rs. Interestingly, we were able to modulate GABA_B_R currents following chronic treatment of OSCs with CGP, but not COR-758. Together, our data fail to reject our null hypothesis and question whether GABA_B_R and Kir3 channel expression is actually altered in APP/PS1 mice.

### GABA_B_R signaling is largely unaffected by aging in the APP/PS1 mouse model of amyloidopathy

A number of recent studies have indicated that GABA_B_Rs (Martín-Belmonte et al., 2020a,b, 2022), their effector Kir3 channels (Martín-Belmonte et al., 2022), and VGCCs (Martín-Belmonte et al., 2025) may display impairment or age-dependent loss in the APP/PS1 mouse model of amyloidopathy. Our physiology, pharmacology, and proteomic data do not support these conclusions. Specifically, aging in the APP/PS1 mouse was not associated with reductions in GABA_B_R/Kir3 signaling—which would be expected from previous electron microscopy analysis (Martín-Belmonte et al., 2022). Indeed, the current density of GABA_B_R signaling, generated by using membrane capacitance (Degro et al., 2024) indicated no change in postsynaptic receptor expression between genotypes at any age. We did observe a functional reorganization of GABA_B_R currents, with the ratio of *str. L-M* to *str. radiatum* GABA_B_R-mediated IPSCs lower in APP/PS1 mice, with WT mice displaying a ratio expected from previous data (Degro et al., 2015). Likewise, GABA_B_Rs have been shown to preferentially localize to dendritic spines (Kulik et al., 2003), a pattern that we observe in our synaptosome preparation—with enriched expression of GABA_B1_ subunits in synaptic fractions compared with total homogenates, but without genotype-specific effects. Interestingly, our physiological and pharmacological findings contradict earlier proteomic (Salazar et al., 2021) and electron microscopy studies (Martín-Belmonte et al., 2020a,b, 2022) which showed overall loss of GABA_B_Rs with progression of Aβ pathology in the APP/PS1 mice. An explanation for divergent GABA_B_R protein levels detected using Western blot in our study may be due to the enrichment of neuron-specific protein in the synaptosomal fraction, which may overlook changes in non-neuronal cells (e.g., microglia and astrocytes) with respect to GABA_B_R expression (Osse et al., 2023). Why our current data deviate from highly detailed electron microscopy assay remains uncertain. With the overexpression of APP in APP/PS1 mice, it is plausible that APP could mask the GABA_B1_ epitope and limit antibody binding, due to the suggested interactions of APP with the extracellular domain of GABA_B1_ subunits (Schwenk et al., 2016; Rice et al., 2019). Such an explanation may also explain the disparity between proteomic and anatomical studies.

However, our data reveal very consistent and robust alterations of GABA_B_R-mediated IPSCs in* str. L-M* relative to *str. radiatum*. This redistribution of evoked receptor currents may reflect a reorganization of GABA_B_R signaling or perhaps a loss of inhibitory synapses in distal dendritic compartments, such as those expressing somatostatin, which has been observed in APP/PS1 mice and postmortem AD brain tissue (Ramos et al., 2006; Schmid et al., 2016; Waller et al., 2020). How GABA_B_R signaling mediated by somatostatin INs is affected in APP/PS1 mice remains unexplored. Another explanation for these discrepancies may be the interaction between APP and GABA_B_Rs at the plasma membrane (Rem et al., 2023), which may plausibly mask epitopes required for EM localization studies. These changes occur against the backdrop of the expected age-dependent accumulation of Aβ plaques in APP/PS1 mice, which begin to appear ∼6 months of age and proliferate up to 1 year of age. However, we observe effects on pre- and postsynaptic GABA_B_R signaling in APP/PS1 mice from 1 month. Given the APP/PS1 mouse overexpresses APP and PS1 over the whole lifespan (Trinchese et al., 2004), this could accelerate phenotypes that would typically be seen with progressed pathology and aging in amyloidopathy. In particular, as APP has been suggested to interact with GABA_B_Rs, this may alter signaling earlier in the disease pathogenesis. Further studies should investigate the localization and function of GABA_B_Rs in other models of dementia—e.g., knock-in APP^N-L-GF^ or 3xTG mouse models to account for such confounds (Sasaguri et al., 2017).

### Fundamental insights into GABA_B_R signaling in development and aging

Stepping back from our main hypothesis, our study provides a rich dataset into the maturation of GABA_B_R signaling in the mammalian hippocampus. Previous studies have indicated that GABA_B_R and Kir3 channel expression undergo rapid postnatal remodeling (López-Bendito et al., 2004). Indeed, it has recently been reported that GABA_B_R postsynaptic currents undergo maturation in the neocortex from 1 to 12 months of age in rats, with baclofen current densities reducing in L2/3 cells and remaining largely stable in L5 neurons (Wilson et al., 2025). Our data suggest that CA1 pyramidal cells more closely match L5 neurons in this instance, with no overt change in current density seen in WT mice from 1- to 12 months of age. Conversely, we find that presynaptic GABA_B_R-mediated inhibition of excitatory neurotransmission becomes weaker with age, while inhibition of inhibition remains strong throughout life. This has important ramifications for our understanding of how neuronal circuits develop throughout life and how local inhibition can shape information transfer. Indeed, it has been shown that baclofen administration in vivo to aged rats leads to slower recruitment of delta-band activity (Fu et al., 2011), which has been recently examined in APP/PS1 mice, where baclofen administration produced diminished delta oscillation enhancement compared with WTs (Yuan et al., 2024) using a putative presynaptic concentration of the drug (Dugladze et al., 2013). In contrast, we found no evidence of GABA_B_R modulation of gamma oscillations in APP/PS1 mice. These studies, combined with our current data, suggest a more prominent role for presynaptic GABA_B_R inhibition, rather than changes in postsynaptic, in both amyloidopathy, and nonpathological aging more generally.

### Pharmacological manipulation of GABA_B_Rs does not differentially affect CA1 pyramidal cell function in APP/PS1 slice cultures

Previous work has indicated that OSCs of mouse models of dementia may display accelerated synaptic dysfunction (Harwell and Coleman, 2016). The data we present here suggest that while APP/PS1 slice cultures may display a hypoexcitability phenotype, in contrast to typical hyperexcitability phenotypes seen in amyloidopathy (Harris et al., 2020; Meftah and Gan, 2023), we see limited evidence for genotype-dependent changes in synaptic function in hippocampal slice cultures from these mice, in broad agreement with similar experiments in previous primary dissociated cell cultures of APP/PS1 mice (Priller et al., 2009; Martinsson et al., 2022). These data suggest that perhaps 6 weeks in vitro may not be sufficient to observe changes in synaptic function in the APP/PS1 mouse, but more nuanced approaches may be required to detect early changes (Harwell and Coleman, 2016; Durrant et al., 2020). Similar to our ex vivo slice data, we found no evidence for altered GABA_B_R signaling in either pre- or postsynaptic domains of CA1 pyramidal cells in slice cultures of APP/PS1 mice. Somewhat surprisingly, we observed no change in presynaptic GABA_B_R-mediated inhibition (which we observed consistently in acute slices), which requires further targeted investigation.

One unexpected finding was the differential impact of chronic GABA_B_R modulation. CGP-55,845 (an orthosteric receptor antagonist) versus COR758 (a NAM; Porcu et al., 2021) displayed divergent effects on receptor-mediated currents. While CGP significantly reduced baclofen-mediated currents and therefore GABA_B_R-mediated currents, this was not seen in COR758 treatment—both of which occurred after washout of the treatments. These data are in line with the potential mechanism of COR758 binding to allosteric domains of GABA_B_Rs, whereas CGP as an orthosteric antagonist (Shaye et al., 2021) may be expected to contribute to receptor desensitization and internalization (Fritzius et al., 2017; Li et al., 2020). Further investigation is required into the mechanisms of such loss of agonist potency.

### Technical limitations

Our conclusions, while consistently failing to reject the null hypothesis, are not without reproach. Notably, due to technical reasons, we were unable to obtain data from >4 mice at 6 months of age for some presynaptic experiments. Notwithstanding, given that in this small sample we find no evidence for alteration of GABA_B_R signaling differing from 1 or 12 months of age, further aging of mice to add additional data would be considered unethical given the other lines of evidence we provide. Second, a major consideration with measuring currents in distal dendrites, such as in *str. L-M* is attenuation of current signals due to issues of space clamp (Degro et al., 2015). Our experiments were not designed to model such potential influences, but given the observed similar CA1 pyramidal cell excitability, we would predict age-dependent changes in GABA_B_R function (if present) to be preserved.

## Conclusions

In summary, we provide evidence that functional GABA_B_R signaling is not substantially reduced following the emergence of Aβ pathology in APP/PS1 mice. Differences in receptor signaling that we observe are present throughout the lifespan. These data suggest that GABA_B_R signaling may be an early subtle change that, therefore, may not be a suitable target in progressed amyloidopathy stages of dementia.

## Synthesis

Reviewing Editor: Karl Herrup, University of Pittsburgh School of Medicine Decisions are customarily a result of the Reviewing Editor and the peer reviewers coming together and discussing their recommendations until a consensus is reached. When revisions are invited, a fact-based synthesis statement explaining their decision and outlining what is needed to prepare a revision will be listed below. The following reviewer(s) agreed to reveal their identity: Christian Wozny.

Synthesis: eN-NWRGR-0099-23

Title: Preventing age-dependent down-regulation of GABAB receptor signaling in a mouse model of amyloid pathology

In 2023, the authors registered their report aiming to investigate a potential link between GABAB receptor signaling and the progression of Alzheimer's disease (AD) in an APP/PS1 mouse model of AD. The manuscript is well drafted. The experimental design is rigorous, the data presented are clear and support the conclusion. The initial hypothesis was that there was a developmental and synapse-specific downregulation of GABAB receptors in this model and that this down-regulation could be prevented by negative modulation of GABAB receptor activity. The study has now been completed, and the results are mostly negative. Despite this, the initial hypotheses were valid, and the field would be well served by seeing the final results published. Before final approval, however, there are some important alterations to the manuscript that would strengthen it and should be addressed. These revisions can be handled at the level of the Reviewing Editor and should not require a re-review.

As pointed out in an earlier review, it is difficult to culture mouse slices. To allow others to repeat the work, the authors describe their methods in a bit more detail and perhaps highlight how these methods differed from OSCs established from rats?

The number of biological replicates (how many animals) were used and what number of technical replicates (how many neurons) were recorded from should be more prominently stated, as well as whether the authors reconstructed neurons from the OSCs?

The relationship of their spatial proximity of the neurons to Aß plaques (lines 315-318) should be illustrated with photomicrographs and provided with a bit more quantitative detail. Were the authors able to examine plaques or Aβ in OSCs?

The writing of the abstract could be clarified, and the absence of positive results explicitly mentioned.

The authors use Figure1D-F to illustrate morphological changes of recorded neurons. To begin the authors should provide more details on how the plaque load was calculated. How were the areas chosen for analysis? Did the analysis take into account the strength of the Aβ signal or just the area of coverage? While the authors claim to find that with higher plaque load, dendritic length decreased, the illustrations appear to show the opposite at both 6-month and 12-month, the APP/PS1 neurons (right, red neurons) have measurably longer expansion and area coverage than the WT neurons (black ones on the left).

In the Discussion, the authors could make more of an effort to offer a mechanistic explanation as to why their initial hypothesis was not supported. The initial logic was sound, so what molecular interactions (or lack thereof) would explain why a known neuromodulator such as Aβ pathology did not affect GABABR signaling. This is particularly true since other authors have suggested significant alterations in GABABR expression and signaling in APP/PS1 mice correlated with Aβ pathology. (Osse and Kinney, 2023 - PMID: 36906075; as well as Salazar et al, 2021 - PMID: 33232936). These data are not contradictory but would offer important context for the Discussion

Minor comments:

In Figures 4 A, C, E and G, the authors should use a consistent scale for the Y-axis to allow easier comparisons among the various recordings. It does not appear that this change would diminish the impact of the results.

Some elaboration of the western blots of Kir3.2 should be offered. The expected band size would be 42-45 kDa (Line 407-408) but in Fig. 2A the band size is marked as a 55kDa band.

The APP/PS1 dendritic segments illustrated for Str. L-M dendrites and Str. Rad dendrites appear visibly thinner than those from the WT at the three ages. Was this a consistent finding and if so would some comment be in order?

Line 160 - There is a space missing between 300 and µm.

Line 1095 - The reference to Meftah and Gan seems incomplete.

The function for for the linear regression analysis done in Fig. 1F should be provided.

## Author Response

Your revision must include the manuscript with new text indicated in a bold or highlighted font to aid the re-review of the manuscript. Separately, please provide a clean copy of the manuscript that includes the title page, which will be published if accepted. Please closely review your manuscript at this time for any final corrections in style or substance. Please consult our Revised Submission Checklist for full details on preparing your revision: https://www.eneuro.org/content/information-authors#revisions All authors are invited to submit visually appealing, high-quality images to be used to promote their article, including on the journal homepage. Please submit your images, as well as an image caption and credit information, directly to the peer review system with your revision. Additional details on sizing and image specifications can be found in the Information for Authors: https://www.eneuro.org/content/information-authors#cover-art Synthesis of Reviews:

Synthesis Statement for Author (Required):

Synthesis: eN-NWRGR-0099-23 Title: Preventing age-dependent down-regulation of GABAB receptor signaling in a mouse model of amyloid pathology In 2023, the authors registered their report aiming to investigate a potential link between GABAB receptor signaling and the progression of Alzheimer's disease (AD) in an APP/PS1 mouse model of AD. The manuscript is well drafted. The experimental design is rigorous, the data presented are clear and support the conclusion. The initial hypothesis was that there was a developmental and synapse-specific downregulation of GABAB receptors in this model and that this down-regulation could be prevented by negative modulation of GABAB receptor activity. The study has now been completed, and the results are mostly negative. Despite this, the initial hypotheses were valid, and the field would be well served by seeing the final results published. Before final approval, however, there are some important alterations to the manuscript that would strengthen it and should be addressed. These revisions can be handled at the level of the Reviewing Editor and should not require a re-review.

We thank the reviewers and editor for their positive appraisal of our manuscript and research performed for this stage 2 manuscript. We agree with their assessment, that while largely negative, these data are relevant for the field. We provide a point-by-point response to the reviewers' comments below.

As pointed out in an earlier review, it is difficult to culture mouse slices. To allow others to repeat the work, the authors describe their methods in a bit more detail and perhaps highlight how these methods differed from OSCs established from rats? While rat organotypic slice cultures have been established for longer, the use of mice to prepare equivalent cultures has been established now for over 10 years, by ourselves (see https://doi.org/10.1186/s13024-016-0110-7, https://doi.org/10.1186/s12974-019-1490-8, https://doi.org/10.1038/s41419-020-2288-4, doi.org/10.1007/978-1-0716-0585-1_10, https://doi.org/10.1186/s40478-021-01141-6, https://doi.org/10.1007/s00401-023-02667-w) and others (for example: https://doi.org/10.1038/s41531-022-00434-4, https://doi.org/10.1007/s00401-021-02264-9,https://doi.org/10.1084/jem.20182184,https://doi.org/10.1186/s13024-019-0346-0, https://doi.org/10.1152/jn.00178.202, doi: 10.1186/s13024-021-00471-2, https://doi.org/10.1038/srep14624, https://pubmed.ncbi.nlm.nih.gov/31392682/).

The purpose of this study was not to perform comparison with rat slice cultures methods, which has been discussed previous. To address the reviewers concerns about reproducibility we have now added extended method detail (lines 232 - 251) The number of biological replicates (how many animals) were used and what number of technical replicates (how many neurons) were recorded from should be more prominently stated, as well as whether the authors reconstructed neurons from the OSCs? We have now added specific numbers of animals and cells to all figure legends. Where subsets of mice have been sampled (Figure 2D-2G) the location of mouse numbers of figures has been indicated.

The relationship of their spatial proximity of the neurons to Aß plaques (lines 315-318) should be illustrated with photomicrographs and provided with a bit more quantitative detail. Were the authors able to examine plaques or Aβ in OSCs? We have now updated figure 1 to better convey the localisation of recorded neurons to plaques present in the APP/PS1 mice at each age, by overlaying neuronal structure with Amyloid-beta labelling. Regarding the presence of plaques in OSCs, we have never observed plaque formation in OSCs from postnatal mice in our hands. We show an example image from a 6-week old APP/PS1 slice culture labelled for Amyloid-beta (MOAB, clone 6C3, Millipore, green), with respect to DAPI (blue) and a recorded neuron (magenta).

This is in line with our previous work that whilst there was accumulation of intraneuronal Amyloid-beta observed over time, plaques did not develop in organotypic slice cultures from TgCRND8 mice, even when cultured for several months (see: https://doi.org/10.1186/s13024-016-0110-7), which is consistent with the findings of others, although increased APP and amyloid-beta were detected (see: https://doi.org/10.1038/cddis.2017.97) The writing of the abstract could be clarified, and the absence of positive results explicitly mentioned.

We have now revised the abstract to reflect the findings of the study. We were previously unclear if this could change from the stage 1 manuscript.

The authors use Figure1D-F to illustrate morphological changes of recorded neurons. To begin the authors should provide more details on how the plaque load was calculated. How were the areas chosen for analysis? Did the analysis take into account the strength of the Aβ signal or just the area of coverage? While the authors claim to find that with higher plaque load, dendritic length decreased, the illustrations appear to show the opposite at both 6-month and 12-month, the APP/PS1 neurons (right, red neurons) have measurably longer expansion and area coverage than the WT neurons (black ones on the left).

We have now updated the methods to include further detail on how plaque load was calculated (lines 439 - 446). Regarding the distribution of dendritic lengths, the example images shown were perhaps the most representative of either the Sholl distribution or dendritic lengths more broadly. We have provided more representative example images to account for this. We have also measured the WT dendritic lengths at 12-months and included these in Figure 1F, to allow more direct comparison between genotypes.

In the Discussion, the authors could make more of an effort to offer a mechanistic explanation as to why their initial hypothesis was not supported. The initial logic was sound, so what molecular interactions (or lack thereof) would explain why a known neuromodulator such as Aβ pathology did not affect GABABR signaling. This is particularly true since other authors have suggested significant alterations in GABABR expression and signaling in APP/PS1 mice correlated with Aβ pathology. (Osse and Kinney, 2023 - PMID: 36906075; as well as Salazar et al, 2021 - PMID: 33232936). These data are not contradictory but would offer important context for the Discussion We have now provided further discussion in lines 788 - 796 providing further discussion of possible explanations for our alternative findings.

Minor comments:

In Figures 4 A, C, E and G, the authors should use a consistent scale for the Y-axis to allow easier comparisons among the various recordings. It does not appear that this change would diminish the impact of the results.

These example traces were amplitude scaled to allow visualisation of the baclofen-mediated inhibition (grey and pink traces). Plotting all example traces on identical scales would lead to a huge range in EPSC sizes within the figure. As all analysis was based on the normalised EPSC post baclofen administration (panels B, D, F, H) the current organisation best reflects the analysis.

Some elaboration of the western blots of Kir3.2 should be offered. The expected band size would be 42-45 kDa (Line 407-408) but in Fig. 2A the band size is marked as a 55kDa band.

The labelling of the Kir3.2 band in Figure 2A was a typographical error, which should have read ~45 kDa. We have now corrected this. We provide below the raw blot images to confirm the position of the Kir3.2 band with respect to the ladder, which was intermediate to the 37 kDa and 50 kDa ladder positions The APP/PS1 dendritic segments illustrated for Str. L-M dendrites and Str. Rad dendrites appear visibly thinner than those from the WT at the three ages. Was this a consistent finding and if so would some comment be in order? We did not test for thickness of dendrites, which would not be an appropriate analysis using the imaging methodology we used (diffraction limited confocal microscopy). However, qualitatively we observed a variety of dendritic thicknesses which was present in both genotypes and ages. We have provided new example images with more consistent dendritic thickness.

Line 160 - There is a space missing between 300 and µm.

This is now corrected Line 1095 - The reference to Meftah and Gan seems incomplete.

This is now corrected, as per the information available from the journal.

The function for for the linear regression analysis done in Fig. 1F should be provided.

This has been added
